# 3p-C-NETA: A versatile and effective chelator for development of Al^18^F-labeled and therapeutic radiopharmaceuticals

**DOI:** 10.7150/thno.75336

**Published:** 2022-08-08

**Authors:** Stephen Ahenkorah, Erika Murce, Christopher Cawthorne, Jessica Pougoue Ketchemen, Christophe M. Deroose, Thomas Cardinaels, Yann Seimbille, Humphrey Fonge, Willy Gsell, Guy Bormans, Maarten Ooms, Frederik Cleeren

**Affiliations:** 1 NURA, Belgian Nuclear Research Center (SCK CEN), Mol, Belgium.; 2 Radiopharmaceutical Research, Department of Pharmaceutical and Pharmacological sciences, University of Leuven, Leuven, Belgium.; 3 Department of Radiology and Nuclear Medicine, Erasmus MC, Rotterdam, the Netherlands.; 4 Nuclear Medicine and Molecular Imaging, Department of Imaging and Pathology, University of Leuven, Leuven, Belgium.; 5 Department of Medical Imaging, College of Medicine, University of Saskatchewan, Saskatoon, Canada.; 6 Department of Chemistry, University of Leuven, Leuven, Belgium.; 7 Life Sciences Division, TRIUMF, Vancouver, Canada.; 8 Department of Medical Imaging, Royal University Hospital (RUH), Saskatoon, Canada.; 9 Biomedical MRI/MoSAIC, Department of Imaging and Pathology, Biomedical Sciences Group, University of Leuven, Leuven, Belgium.

**Keywords:** 3p-*C*-NETA, Al^18^F, PET, targeted radionuclide therapy, radiopharmaceutical

## Abstract

**Background**: Radiolabeled somatostatin analogues (*e.g.* [^68^Ga]Ga-DOTATATE and [^177^Lu]Lu-DOTATATE) have been used to diagnose, monitor, and treat neuroendocrine tumour (NET) patients with great success. [^18^F]AlF-NOTA-octreotide, a promising ^18^F-labeled somatostatin analogue and potential alternative for ^68^Ga-DOTA-peptides, is under clinical evaluation. However, ideally, the same precursor (combination of chelator-linker-vector) can be used for production of both diagnostic and therapeutic radiopharmaceuticals with very similar (*e.g.* Al^18^F-method in combination with therapeutic radiometals ^213^Bi/^177^Lu) or identical (*e.g.* complementary Tb-radionuclides) pharmacokinetic properties, allowing for accurate personalised dosimetry estimation and radionuclide therapy of NET patients. In this study we evaluated 3p-*C*-NETA, as potential theranostic Al^18^F-chelator and present first results of radiosynthesis and preclinical evaluation of [^18^F]AlF-3p-*C*-NETA-TATE.

**Methods**: 3p-*C*-NETA was synthesized and radiolabeled with diagnostic (^68^Ga, Al^18^F) or therapeutic (^177^Lu, ^161^Tb, ^213^Bi, ^225^Ac and ^67^Cu) radionuclides at different temperatures (25-95 °C). The *in vitro* stability of the corresponding radiocomplexes was determined in phosphate-buffered saline (PBS) and human serum. 3p-*C*-NETA-TATE was synthesized using standard solid/liquid-phase peptide synthesis. [^18^F]AlF-3p-*C*-NETA-TATE was synthesized in an automated AllinOne® synthesis module and the *in vitro* stability of [^18^F]AlF-3p-*C*-NETA-TATE was evaluated in formulation buffer, PBS and human serum. [^18^F]AlF-3p-*C*-NETA-TATE pharmacokinetics were evaluated using µPET/MRI in healthy rats, with [^18^F]AlF-NOTA-Octreotide as benchmark.

**Results**: 3p-*C*-NETA quantitatively sequestered ^177^Lu, ^213^Bi and ^67^Cu at 25 °C while heating was required to bind Al^18^F, ^68^Ga, ^161^Tb and ^225^Ac efficiently. The [^18^F]AlF-, [^177^Lu]Lu- and [^161^Tb]Tb-3p-*C*-NETA-complex showed excellent *in vitro* stability in both PBS and human serum over the study period. In contrast, [^67^Cu]Cu- and [^225^Ac]Ac-, [^68^Ga]Ga-3p-*C*-NETA were stable in PBS, but not in human serum. [^18^F]AlF-3p-*C*-NETA-TATE was obtained in good radiochemical yield and radiochemical purity. [^18^F]AlF-3p-*C*-NETA-TATE displayed good *in vitro* stability for 4 h in all tested conditions. Finally, [^18^F]AlF-3p-*C*-NETA-TATE showed excellent pharmacokinetic properties comparable with the results obtained for [^18^F]AlF-NOTA-Octreotide.

**Conclusions**: 3p-*C*-NETA is a versatile chelator that can be used for both diagnostic applications (Al^18^F) and targeted radionuclide therapy (^213^Bi, ^177^Lu, ^161^Tb). It has the potential to be the new theranostic chelator of choice for clinical applications in nuclear medicine.

## Introduction

Apart from [^18^F]FDG and [^18^F]NaF, other positron emission tomography (PET) radiopharmaceuticals that are frequently used in clinical practice are chelator-based ^68^Ga-labeled radiopharmaceuticals such as [^68^Ga]Ga-PSMA-11 and [^68^Ga]Ga-DOTATATE for imaging of prostate cancer and neuroendocrine tumors (NETs), respectively 1,2. However, in recent years, alternatives to ^68^Ga-labeled tracers have been developed, including ^18^F-labeled radiopharmaceuticals, as fluorine-18 offers several logistic advantages over gallium-68. Fluorine-18 can be produced by cyclotrons in large amounts, which is not the case for ^68^Ga if a ^68^Ge/^68^Ga-generator is used 3. This disadvantage could be remedied by advances in the cyclotron production of gallium-68, however, this method may not be feasible for all cyclotron facilities 4. The relatively longer half-life of ^18^F (T_1/2_: 109.8 min) compared to ^68^Ga (T_1/2_: 68 min) allows delayed imaging procedures, which might improve the rate of lesion detection, and allows fluorine-18-labeled tracers to be transported to PET centers at long distance from the production site. Consequently, production and quality control of ^18^F-labeled radiopharmaceuticals can be centralized and shipped to remote hospitals that do not have a cyclotron or radiopharmacy on site 3. Finally, fluorine-18 has a shorter positron range than gallium-68, which can contribute to higher spatial resolution on the newest generation of clinical PET scanners.

**Figure 1:**
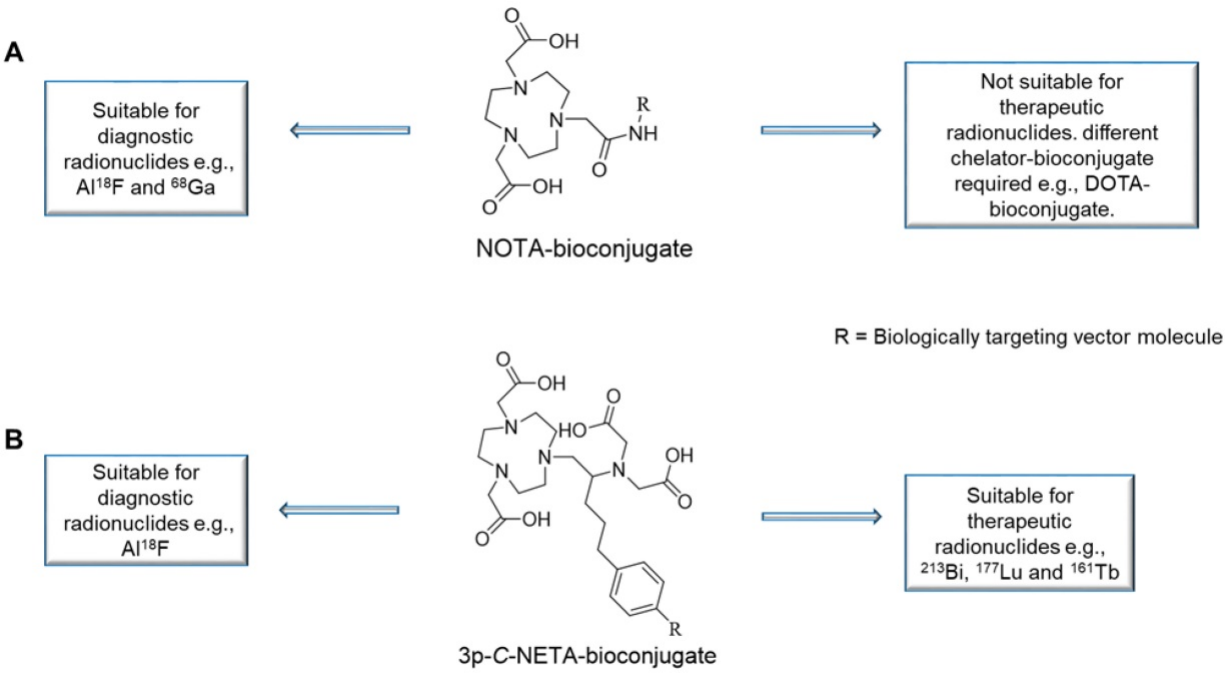
** Versatility of 3p-C-NETA in contrast to NOTA.** (**A):** NOTA is only suitable for radiolabeling of diagnostic radionuclides (Al^18^F and ^68^Ga). (**B):** In contrast, 3p-C-NETA has potential for both diagnostic (Al^18^F) and therapeutic radionuclides (^213^Bi, ^177^Lu, and ^161^Tb), making it a true theranostic chelator.

The Al^18^F-method combines the advantages of a chelator-based radiolabeling method with the imaging and logistical advantages of fluorine-18. [^18^F]AlF-NOTA-Octreotide and [^18^F]AlF-FAPI-74 are some excellent examples of this exciting and promising development 5-7. A major drawback is that the most utilized chelators for the Al^18^F-method, 1,4,7-triazacyclononane-*N,N',N''*-triacetic acid (NOTA, **Figure 1A**), a pentadentate ligand when conjugated to a vector molecule since one of the of the three carboxylic arms is used for amide bond formation, is not compatible with therapeutic radionuclides such as the β^-^-emitter lutetium-177 (^177^Lu) and the promising α-emitters actinium-225 (^225^Ac) and bismuth-213 (^213^Bi). Therefore, two different precursors are required to produce the diagnostic and therapeutic radiopharmaceuticals. 1,4,7,10-tetraazacyclododecane-1,4,7,10-tetraacetic acid (DOTA) remains the most utilized chelator for therapeutic radionuclides such as ^177^Lu, ^225^Ac and ^213^Bi despite that several drawbacks of this chelator have been identified. The classic radiolabeling conditions for DOTA involves heating at high temperatures (*e.g.*, 30-60 min at 95 °C), which is not compatible with heat-sensitive vector molecules. Furthermore, it has been shown that high concentrations of DOTA-ligands are required to achieve quantitative yields due to poor kinetic properties 8,9.

To have a real theranostic pair allowing accurate, personalised dosimetry estimation for radionuclide therapy, the pharmacokinetics of the diagnostic and therapeutic radiopharmaceutical should be very similar or identical 10. Therefore, it might be an advantage that the same precursor (combination of chelator-linker-vector) is used for the production of both the diagnostic and therapeutic radiopharmaceutical with very similar (*e.g.* Al^18^F-method in combination with therapeutic radiometals ^213^Bi/^177^Lu) or identical (*e.g.* complementary Tb-radionuclides) pharmacokinetic properties. The role of predictive personalized dosimetry in clinical practice is still limited however, and further preclinical and clinical studies are required to evaluate the benefits of personalized dosimetry using a theranostic pair. Another advantage of using the same precursor molecule for production of the diagnostic and therapeutic radiopharmaceutical is that only one GMP-grade precursor molecule needs to be developed and produced, resulting in an economical advantage and reducing the development time.

To address these problems, we decided to look for an alternative to NOTA as Al^18^F-chelator that also can be used in combination with therapeutic radionuclides, and has superior labeling kinetics over DOTA, which is especially important in combination with the short-lived α-emitting radionuclide ^213^Bi. Indeed, because of the high cost of the α-emitting radionuclides, short physical half-life in the case of ^213^Bi and radioprotection requirements, quantitative yields using fast and mild (automated) radiolabeling conditions are desired to facilitate efficient GMP-compliant on-site production of ^213^Bi radiopharmaceuticals.

**Figure 2:**
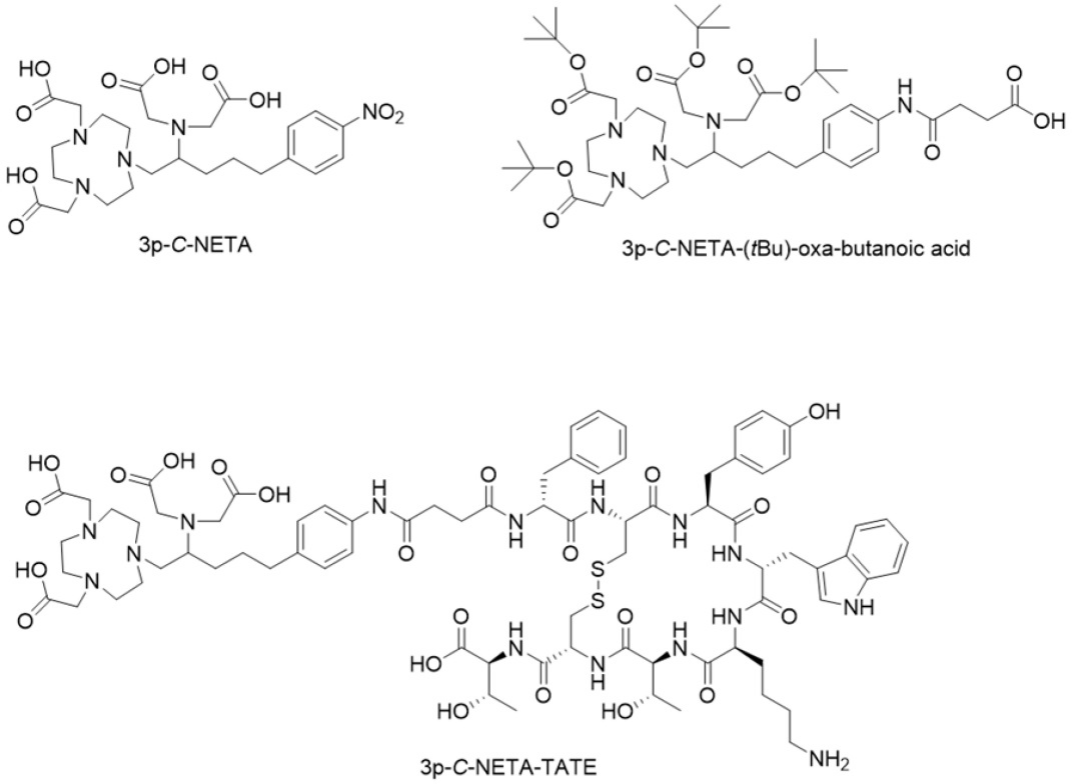
Chemical structures of 3p-*C*-NETA, 3p-*C*-NETA-(*t*Bu)-oxa-butanoic acid and 3p-*C*-NETA-TATE.

3p-*C*-NETA ({4-[2-(bis-carboxy-methylamino)-5-(4-nitrophenyl)-entyl]-7-carboxymethyl-[1,4,7]tri-azonan-1-yl} acetic acid; **Figure 2**) 11-13, a ligand possessing both a parent macrocyclic NODA (1,4,7-triazacyclononane-*N,N'*-diacetic acid) backbone and a flexible acyclic tridentate pendant arm, has been reported by Chong *et al.* to be a promising chelator in terms of kinetics and stability for β^—^emitters, such as ^90^Y and ^177^Lu 11. 3p-*C*-NETA instantly (< 5 min) formed a complex with ^90^Y and ^177^Lu (>95%) 14. Stimulated by these interesting results, Kang *et al.* evaluated the *in vitro* stability and pharmacokinetic properties of ^90^Y and ^177^Lu-labeled 3p-*C*-NETA-trastuzumab in tumor bearing mice, with excellent results 11. Recently, Cassells *et al.* also identified 3p-*C*-NETA as an ideal chelator for terbium radionuclides, such as the promising therapeutic radionuclide terbium-161 15. Further, exceptional labeling kinetics with ^205/6^Bi and stability studies of the resulting Bi-complex were reported, indicating that 3p-*C*-NETA could be an ideal chelator for ^213^Bi 12. As 3p-*C*-NETA also contains a NODA backbone, we envisaged that it might be a suitable chelator for Al^18^F-chelation and thus could be a true theranostic chelator for ^18^F and therapeutic radionuclides (**Figure 1B**), as DOTA is for ^68^Ga and ^177^Lu.

In this study we present the synthesis of the 3p-*C*-NETA, based on a published method 16, and its new bifunctional derivative 3p-*C*-NETA-(*t*Bu)-oxa-butanoic acid (**Figure 2**). Next, we evaluated the radiolabeling properties of 3p-*C*-NETA with a range of therapeutic (^213^Bi, ^161^Tb, ^177^Lu, ^67^Cu, and ^225^Ac) and diagnostic radionuclides (Al^18^F and ^68^Ga) followed by *in vitro* stability studies in our search for a true theranostic chelator. After obtaining encouraging results, we coupled 3p-*C*-NETA-(*t*Bu)-oxa-butanoic acid to the somatostatin analogue (Tyr^3^)-octreotate to obtain 3p-*C*-NETA-TATE (**Figure 2**) and determined IC_50_ values of 3p-*C*-NETA-TATE, labeled with stable AlF, Lu, Tb and Bi using cell membranes overexpressing human SSTR2. Further, we report here the automated synthesis of [^18^F]AlF-3p-*C*-NETA-TATE, the *in vitro* stability and radiometabolite studies of [^18^F]AlF-3p-*C*-NETA-TATE in rats. Finally, the pharmacokinetics and SSTR2 specificity of [^18^F]AlF-3p-*C*-NETA-TATE was assessed by performing baseline and blocking scans in rats using µPET/MR and results were compared with the established SSTR2 radiopharmaceutical [^18^F]AlF-NOTA-octreotide 6.

## Experimental section

### Materials

Reagents and solvents: All chemicals and solvents were purchased from commercial suppliers such as Sigma-Aldrich (Bornem, Belgium), Fluka (Bornem, Belgium), Fisher (Doornik, Belgium) and Acros Organics (Geel, Belgium) and were used without further purification. *p*-SCN-Bn-DOTA (DOTA) was purchased from Macrocyclics, Inc (Plano, Texas, USA). Details regarding the synthesis of 3p-C-NETA-(*t*Bu)-oxa-butanoic acid 16 and 3p-*C*-NETA-TATE 17 can be found in the supplementary data.

### Radiochemistry

All studies involving radionuclides were carried out in laboratories with adequate lead block shielding and designated fume hoods. All water was deionized and passed through Millipore water purification system until a resistivity of 18 MΩ·cm was achieved. Gallium-68 was eluted in the form of [^68^Ga]GaCl_3_ from a commercially available IGG101 Pharmaceutical Grade Generator from Eckert & Ziegler (Berlin, Germany) using 0.1 M HCl. To concentrate gallium-68 and remove any germanium-68 in the generator eluate, the mixture was applied on a Chromafix PS-H^+^ column (Fisher Scientific Oy - Ratastie 2, 01620 Vantaa - Finland) and finally gallium-68 was eluted with 1.5 mL of 5 M sodium chloride into a reactor vial. Fluorine-18 was produced on site using a cyclotron (IBA Cyclone 18/9, IBA, Louvain-la-Neuve, Belgium) by irradiation of H_2_^18^O with 18-MeV protons. [^177^Lu]LuCl_3_ (0.05 M HCl) was purchased from ITM Medical Isotopes GmbH (ITM Group, Garching Munich, Germany). [^161^Tb]TbCl_3_ (0.05 M HCl), [^213^Bi]BiI_5_^2-^ (0.1 M HCl/ 0.1 M NaI) and [^225^Ac]Ac(NO_3_)_3_ (0.5 M HNO_3_) were produced on-site at SCK CEN based on literature 9,18,19, [^67^Cu]CuCl_2_ (0.01 M HCl) was procured from Canadian Isotope Innovations Corp. (Saskatoon, Canada). All radiolabeling buffers were treated with Chelex 100 [sodium form (50-100 mesh, Sigma Aldrich)] for 15 min to remove trace metals. All solutions were degassed and filtered before use. All complexation reactions were performed at 25, 40, 55 or 95 °C for [^68^Ga]GaCl_3_, [^18^F]AlF, [^177^Lu]LuCl_3_ or [^161^Tb]TbCl_3_ except for [^213^Bi]BiI_5_^2-^ and [^225^Ac]Ac(NO_3_)_3_ which were only performed at 25, 55 or 95 °C due to limited availability of the radionuclide and at 37 or 95 °C for [^67^Cu]CuCl_2_. 3p-*C*-NETA was labeled by adding [^68^Ga]GaCl_3_: (5 MBq, 0.5 M NaOAc, pH 5.2); [^18^F]AlF: (4 MBq, 0.1M NaOAc, pH 4.1); [^161^Tb]TbCl_3_: (4 MBq, 0.1M NaOAc, pH 4.1); or [^177^Lu]LuCl_3_: (6 MBq, 0.1M NaOAc, pH 4.1) to the 3p-*C*-NETA solution (10 µM or 150µM for Al^18^F-labeling), in the corresponding buffer solution and the reaction mixture (V= 1 mL) was incubated for 12 min at the desired temperature.

^213^BiI_5_^2-^ and [^225^Ac]Ac(NO_3_)_3_: The radiolabeling experiments were performed by reacting 200-220 kBq ^213^BiI_5_^2-^ with different concentrations of 3p-*C*-NETA (5, 10, 20 and 50 µM in 0.37 M TRIS buffer, pH 8.5, V = 300 µL) with DOTA as a benchmark and radiochemical conversion (RCC) was determined at 5, 10 and 15 min. Similar reaction conditions were followed for [^225^Ac]Ac(NO_3_)_3_, however, 90-100 kBq of ^225^Ac was used with RCC determination at 1 h or 2 h. [^67^Cu]CuCl_2_: The labeling was performed by adding 10 MBq [^67^Cu]CuCl_2_ to 10 µM solution of 3p-*C*-NETA ( 0.15 M NH_4_OAc, pH 5.8, V = 50 µL) and RCC was determined at 30 and 60 min. The RCC was evaluated by instant thin-layer liquid chromatography (iTLC-SG, Varian, Diegem, Belgium). iTLC-SG papers were developed in an elution chamber using acetonitrile : water (75/25 v/v). The retention factor (rf) of free [^68^Ga]GaCl_3_, [^18^F]AlF, [^177^Lu]LuCl_3_, [^161^Tb]TbCl_3_, [^213^Bi]BiI_5_^2-^ and [^225^Ac]Ac(NO_3_)_3_ was 0.14-0.22. The Rf of the radiocomplexes was 0.91-0.94. The distribution of activity on the iTLC chromatograms was analyzed using phosphor storage autoradiography [Perkin Elmer, Waltham, USA processed in a Cyclone Plus system (Perkin Elmer) and Optiquant software (Perkin Elmer)] except for ^213^Bi, ^67^Cu and ^225^Ac where distribution of activity was counted on a gamma counter. Activity of ^225^Ac was counted using the ^213^Bi-peak window (380-500 keV) after a time delay of 24 h to allow ^213^Bi to reach equilibrium with ^225^Ac as described 20.

### *In vitro* stability of 3p-*C*-NETA radiocomplexes

All radio-synthesized complexes were purified with a Sep-Pak C_18_ Light cartridge (Waters, Eschborn, Germany) as previously described 15. Briefly, the Sep-Pak C_18_ Light cartridge was pre-conditioned with absolute ethanol (5 mL) followed by water (5 mL). The reaction mixture was loaded onto the cartridge and washed with 6-8 mL water to remove unbound radionuclide. The pure radio-complex was eluted with 0.25 mL absolute ethanol and the volume was brought to 0.5 mL by diluting with 0.25 mL of 0.9% NaCl. 50 µL of the purified radio-complex was added to a 1 mL vial containing either 450 µL of PBS or human serum and the solution was incubated at 37 °C under constant gentle shaking. To determine the percentage of intact radio-complexes, 5 µL samples were taken for iTLC analysis at selected times points for Al^18^F and ^68^Ga radiocomplexes (10, 30, 60, 120 and 240 min), while ^177^Lu, ^161^Tb and ^225^Ac radiocomplexes were studied up to 10 days (days: d) and ^67^Cu up to 4 d. Analysis of the activity distribution was performed as described above. Stability of [^213^Bi]Bi-3p-*C*-NETA was not tested due to the short half-life and limited availability of ^213^Bi.

### 3p-*C*-NETA-TATE metal complex membrane-based affinity studies

Competition binding studies (IC_50_) were performed as previously described 21 using purified Chinese hamster ovary-K1 (CHO-K1) cell membranes overexpressing human SSTR2 (membrane target system^TM^, Perkin Elmer, Zaventem, Belgium). The assay measures binding affinity by evaluating the ability of the compounds ([^nat^Tb]Tb-3p-*C*-NETA-TATE, [^nat^Bi]Bi-3p-*C*-NETA-TATE, [^nat^Lu]Lu-3p-*C*-NETA-TATE, [^nat^F]AlF-3p-*C*-NETA-TATE with [^nat^F]AlF-NOTA-Octreotide as benchmark) to compete with the radioactive reference ligand [^111^In]In-DOTA-TATE for SSTR2 binding sites on CHO-K1 membranes (see supplementary materials for detailed synthesis of the reference compounds and IC_50_ study protocol). IC_50_ values were calculated using nonlinear regression (four parameters) using GraphPad Prism 9 (Graph Pad Software, San Diego, CA, USA) based on three independent biological replicates for each concentration tested.

### Radiosynthesis of [^18^F]AlF-3p-*C*-NETA-TATE, QC system and *in vitro* stability

[^18^F]AlF-3p-*C*-NETA-TATE was synthesized in an automated AllinOne® synthesis module (Trasis, Liège, Belgium) using 3p-*C*-NETA-TATE as precursor and by using the identical protocol as described by Tshibangu *et al.* for the radiosynthesis of [^18^F]AlF-NOTA-Octreotide 22. High pressure liquid chromatography (a Shimadzu LC20A HPLC System, wavelength = 220 nm, Kyoto, Japan) coupled in series to a DAD-UV detector and a shielded 3-inch NaI (Tl) scintillation detector connected to a single channel analyzer (Gabi box, Elysia-Raytest, Straubenhardt, Germany) was used for the identification and determination of radiochemical purity (RCP) of [^18^F]AlF-3p-*C*-NETA-TATE**.** The recovery of [^18^F]F^-^ and [^18^F]AlF is >95% on this system, as previously determined by Tshibangu *et al.* 22,23. HPLC column: C_18_ column (Waters XBridge® 3.5 µm, 3.0 x 100 mm, 0.8 mL/min flowrate); mobile phase composition: A (0.05 M ammonium acetate, pH = 5.5): 0-5 min (95%), 5-25 min (80 → 75%), 25.1-30 min (95%); B (acetonitrile): 0-5 min (5%), 5-25 min (20 → 25%), 25.1-30 min (5%). To determine the *in vitro* stability, 5 µL (2 MBq in EtOH/NaAsc 0.59% in NaCl 0.9% 1.1/11.9 V/V in water) of [^18^F]AlF-3p-*C*-NETA-TATE was added to a 1 mL vial containing either 495 µL of PBS, human serum or formulation buffer (EtOH/NaAsc 0.59% in NaCl 0.9% 1.1/11.9 V/V in water) at 37 °C with constant shaking. To determine the percentage of intact tracer at selected times points (10, 30, 60, 120 and 240 min) radio-HPLC was performed as described above.

### Animals

Female Wistar rats were housed in individually ventilated cages in a thermo-regulated (~22 °C), humidity-controlled facility under a 12 h-12 h light-dark cycle, with access to food and water ad libitum. The number of animals included in the study was based on a previous report where a >75% difference was seen between naïve and blocked animals in SSTR2 expressing organs 22; detection of this difference therefore required 3 animals per group (power = 0.95 and α = 0.05) assuming a 20% standard deviation within the groups (calculation made using G*Power 3.1.9.7) 24. All animals included in the study were randomly selected from the pool of rats. There were no exclusion criteria, and all subsequent studies and analyses were conducted unblinded.

### Plasma and urine radiometabolite studies of [^18^F]AlF-3p-*C*-NETA-TATE

Plasma and urine radiometabolite studies were performed as previously reported 22. In brief, Female Wistar Rats (n = 3) were injected with [^18^F]AlF-3p-*C*-NETA-TATE (3-5 MBq) through the tail vein under anesthesia (2.5% isoflurane in O_2_ at 1 L/min flow rate) and the animals were kept under anesthesia throughout the experiment. Urine samples were collected 30 min post injection and filtered through a 0.22 µm filter (Millex-GV, 0.22 µm, PVDF, 13 mm, Merck KGaA, Darmstadt, Germany) before being stored on ice. Blood samples were collected via the tail vein at 10 and 30 min post injection and stored on ice in EDTA-containing tubes (0.5 mL, Greiner Bio-One K_2_EDTA MiniCollect tubes). The tubes were centrifuged at 2333 x g for 10 min, plasma was collected, and the recovery (>95%) was determined with a gamma counter (Perkin-Elmer, Wizard2 2480, Waltham, Massachusetts, USA). As a control, urine and blood samples from non-injected animals were spiked with [^18^F]AlF-3p-*C*-NETA-TATE (0.05-0.5 MBq) and processed using the same procedure. 5 µL of the plasma or urine sample was injected on the radio-HPLC system equipped with a Chromolith performance column (C18, 4.6 mm × 100 mm, Merck KGaA, Darmstadt, Germany). The elution gradient is shown in Table S5 with mobile phase A: ammonium acetate 0.05M pH 5.5 and acetonitrile as mobile phase B.

### *In vivo* pharmacokinetics and biodistribution of [^18^F]AlF-3p-*C*-NETA-TATE

Dynamic PET scans were performed on a small animal PET/MR scanner based on previously reported protocol 22. Briefly, female Wistar rats (208-231 g) were anaesthetized with a 5% isoflurane/ oxygen mixture before being maintained at 1-2% isoflurane/1 L/minute throughout the experiment. Rats tail veins were cannulated before being placed in the imaging cell (Bruker Biospin) which was then placed into the scanner; temperature and respiration were monitored throughout the scanning via a physiological monitoring system (SA Instruments, Stony Brook, NY, USA). Rats were injected with [^18^F]AlF-3p-*C*-NETA-TATE (5.5-18.5 MBq, apparent molar activity 22 ± 8 GBq/μmol) at the start of the PET acquisition in presence or absence of octreotide acetate (2.5 mg/kg co-injection with radiopharmaceutical; blocking scans were performed the following day in the same animals). 75 min scans were carried out with the pituitary to kidneys in the PET FOV, during which respiration-gated 3D spin-echo MRI (TurboRARE sequence, TR = 500 mseconds (ms), TE = 18.8 ms, RARE factor = 8, FOV 14x6x6 cm, isotropic resolution of 0.5 mm) images were acquired with a quadrature radio-frequency resonator (transmit/receive, 86 mm internal diameter, Bruker Biospin). PET and MRI scans were then repeated at 100-120 minutes with the animal in the same position. Dynamic PET data were divided into timeframes (4×15 s, 1×240 s, 7×600 s) with corrections for decay, randoms, scatter and deadtime and then reconstructed using an MLEM algorithm with 24 iterations and an isotropic voxel size of 0.5 mm. PET images were normalized to injected activity and animal weight to give standardized uptake values (SUV), and selected organs were outlined to create volumes of interest after coregistration with MR images of which SUV_mean_ values are reported; a circular VOI was placed over the splenic division of the pancreas (as visualised via tracer uptake and mapped to the blocked scan) to determine pancreatic uptake. All image analysis was carried out using the PFUSIT module of PMOD (v 4.004, PMOD Technologies Ltd., Zurich, Switzerland). Quantitative data are expressed as mean ± standard error of the mean (SEM).

## Results and Discussion

### Synthesis of 3p-*C*-NETA and 3p-*C*-NETA-TATE

As 3p-*C-*NETA is not commercially available, we synthesized 3p-*C*-NETA based on the formation of the *N,N'*-bisubstituted-β-amino iodide and nucleophilic ring-opening of an aziridinium ion, as reported in the literature 16. Briefly, intramolecular rearrangement of β-iodoamine, for formation of the aziridinium ion, followed by regiospecific ring opening with di-*tert*-butyl 2,2'-(1,4,7-triazonane-1,4-diyl)diacetate (S_N_2 pathway) provided the desired product 3p-*C*-NETA-(*t*Bu) in a good isolated yield of 84% (>95% purity) 16. The *t*-butyl groups were removed by the treatment of 3p-*C*-NETA-(*t*Bu) with TFA to afford 3p-*C*-NETA (>96% purity). 3p-*C*-NETA was used as such for initial radiolabeling and *in vitro* stability studies. To enable conjugation of 3p-*C*-NETA to a vector molecule, 3p-*C*-NETA was functionalized initially with isothiocyanate as reported by Kang *et al.* 16*.* However, conjugation to the (Tyr^3^)-octreotate moiety by forming a thiourea bond resulted in a plethora of undesired side products. Therefore, we changed the functionalization strategy by introducing succinic acid to form 3p-*C*-NETA-(tBu)-oxa-butanoic acid (**Figure 2**, see supplementary scheme 1) to enable efficient conjugation via amide bond formation (see supplementary scheme 2). The corresponding 3p-*C*-NETA-TATE conjugate (**Figure 2**, 37% yield) was successfully purified using preparative HPLC (purity >95%).

### Radiolabeling of 3p-*C*-NETA

3p-*C*-NETA has shown great promise as a chelator for β^-^-emitting radionuclides (^177^Lu, ^90^Y, ^161^Tb) 11,15, but has not been evaluated for diagnostic and alpha-emitting radionuclides. Hence, to explore this compound further for potential theranostic applications, we evaluated the labeling properties of 3p-*C*-NETA with a range of diagnostic (Al^18^F and ^68^Ga) and therapeutic radionuclides (^177^Lu, ^161^Tb , ^67^Cu, ^213^Bi and ^225^Ac; see supplementary table 1, 2 and 3).

#### Al^18^F

**Figure 3:**
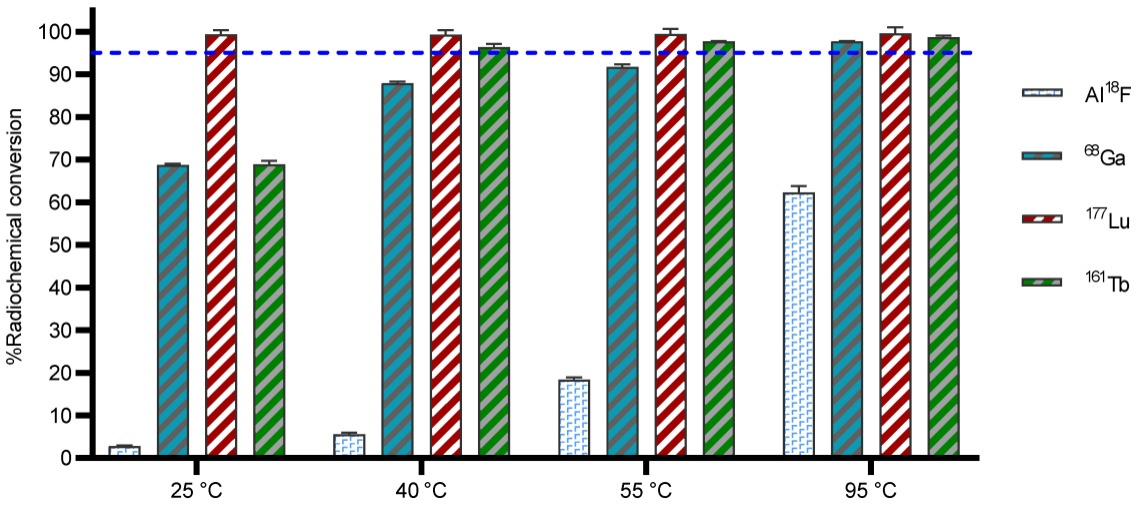
** Radiolabeling efficiency with a range of diagnostic and therapeutic radionuclides using 3p-C-NETA**. 3p-C-NETA (150 µM) was labeled with 4-8 MBq of [^18^F]AlF (0.1 M NaOAc, pH 4.1), while 3p-C-NETA (10 µM) was labeled with [^68^Ga]GaCl_3_ (0.5 M NaOAc, pH 5.2), [^177^Lu]LuCl_3_ (0.1 M NaOAc, pH 4.1) and [^161^Tb]TbCl_3_ (0.1 M NaOAc, pH 4.1) for 12 min at 25, 40, 55 or 95 °C. Blue line is inserted to indicate a yield of 95%.

The Al^18^F labeling method, first reported by McBride *et al.*, 25 allows radiofluorination of vector molecules in aqueous medium in a one-step procedure. This approach avoids time-consuming multistep procedures that are mostly required when using prosthetic groups to radiolabel peptides or biomolecules with fluorine-18 25. Till date, the macrocyclic hexadentate ligand NOTA and especially the pentadentate derivative NODA remains the gold standard for Al^18^F-labeling of heat-stable molecules, but high temperatures (*e.g.*, 95 °C) are needed to obtain good RCCs. 3p-*C*-NETA has the same macrocyclic core (triazacyclononane backbone) as NODA, but has in addition a flexible acyclic tridentate pendant arm (iminodiacetic acid) which was expected to improve the reaction kinetics to coordinate [^18^F]AlF compared to NODA. However, according to Figure 3 and Table S1, the labeling kinetics are obviously noticeably slow reaching RCCs after 12 min reaction time of 2.8 ± 0.2% at 25 °C and 8.8 ± 0.4% at 40 °C, only. raising the temperature to 55 °C resulted in a RCC of 18.4 ± 0.7%. Only at 95 °C, a good RCC of 62.3 ± 1.5% was obtained (**Figure 3, Table S1**). It has been reported before that performing the Al^18^F-reaction at lower ionic strength by addition of co-solvents (*e.g.* ethanol, acetonitrile), increased RCCs considerably 26. Accordingly, a reaction content of 50% absolute ethanol increased the RCC at 95 °C to 80.3 ± 0.6%, and this is comparable to the [^18^F]AlF-labeling of NODA-benzyl 27.

#### ^68^Ga

DOTA derivatives are still the most used chelators for nuclear medicine applications, including ^68^Ga-labeling. This is primarily due to its universal applicability for a wide range of (three-valent) metal ions and the commercial availability of DOTA derivatives, which is based on the fact that lanthanide(III) complexes of DOTA derivatives and their conjugates have been widely used as MRI contrast agents. On the other hand, 1,4,7-triazacyclononane derivatives (*e.g.* NOTA, NODAGA) exhibit much better selectivity towards the Ga^3+^ ion and their complexes are more stable, as the size of the 1,4,7-triazacyclononane cavity is nearly ideal for this ion 28. To determine the versatility of 3p-*C*-NETA, radiolabeling with ^68^Ga was performed. The RCC of ^68^Ga with 3p-*C*-NETA was moderate at 25 °C (68.7 ± 0.7%), however, RCCs of 87.9 ± 0.4% and 91.7 ± 0.7% were observed at 40 and 55 °C, respectively. At 95 °C, an excellent RCC of 97.7 ± 0.1% was observed (**Figure 3, Table S1**).

#### ^177^Lu

The most widely used radiometal for vectorized radionuclide therapy in the clinic is ^177^Lu and DOTA chelators are mostly used to form a theranostic pair with the diagnostic ^68^Ga-DOTA-ligands (*e.g.* [^177^Lu]Lu-DOTATATE and [^68^Ga]Ga-DOTATATE). Despite the fact that DOTA has demonstrated excellent clinical applications with ^177^Lu, slow labeling kinetics such as labeling at elevated temperatures (*e.g.*, 95 °C), high ligand concentration (*e.g.*, 100 µM) and prolonged reaction time (30-60 min) are significant and must not be overlooked 9. Complexation of [^177^Lu]LuCl_3_ by 3p-*C*-NETA was completed in 12 min at 25 °C, as determined by iTLC (RCC of 99.4 ± 0.4%; **Figure 3, Table S1**) and this observation was comparable to literature (RCC of 99.8 ± 0.1, 10 min, RT, 0.25 M NH_4_OAc, pH 5, 100 µg ligand) 11. Similar results were observed at 95 °C. These results confirm the excellent properties of 3p-*C*-NETA as chelator for ^177^Lu.

#### ^161^Tb

The radiolanthanide ^161^Tb has gathered increasing interest in recent years due to its favorable properties for medical applications 29. ^161^Tb has similar chemical and physical properties as ^177^Lu. However, the advantages of ^161^Tb over ^177^Lu are numerous Firstly, more Auger/conversion electrons (energy ≤50 keV) are produced during decay of ^161^Tb than for ^177^Lu. Because of the short range of auger electrons in tissue (subcellular, order of nanometers), they become therapeutically very relevant if they are in close proximity to a radiosensitive molecular target such as the DNA 30. Secondly, Tb-radiopharmaceuticals can be used in a true theranostic approach as there are also diagnostic terbium radionuclides available such as ^152^Tb (β^+^ emitter, T_1/2_: 17.5 h, E_β+_ average = 1.140 MeV) and ^155^Tb (EC γ emitter, T_1/2_: 5.32 d, Eγ = 0.105 MeV) which can be used for PET and single photon emission computed tomography (SPECT) applications, respectively 29. Finally, ^149^Tb ((3.97 MeV, I_α_ = 16.7%) is a promising alpha-emitter which might even improve therapeutic efficacy as combination or stand-alone treatment 30.

A RCC of 68.9 ± 0.8% (**Figure 3**) was observed with 3p-*C*-NETA (10 µM) at 25 °C. However, a RCC of >95% was observed at 40 °C for 3p-*C*-NETA (10 µM), and even at a concentration of 0.1 µM, an excellent RCC (>95%) was observed, which was not the case for DOTA and DOTA-GA, with RCCs of 38% and 35% respectively (reaction conditions: 10 µM ligand, 0.1 M NaOAc, pH 4.7) 15. Therefore, 3p-*C*-NETA seems to be a very promising chelator for the unique matched quadruplet of terbium radioisotopes, including the Tb-labeling of heat-sensitive biomolecules such as antibodies and antibody fragments.

#### ^67^Cu

**Figure 4:**
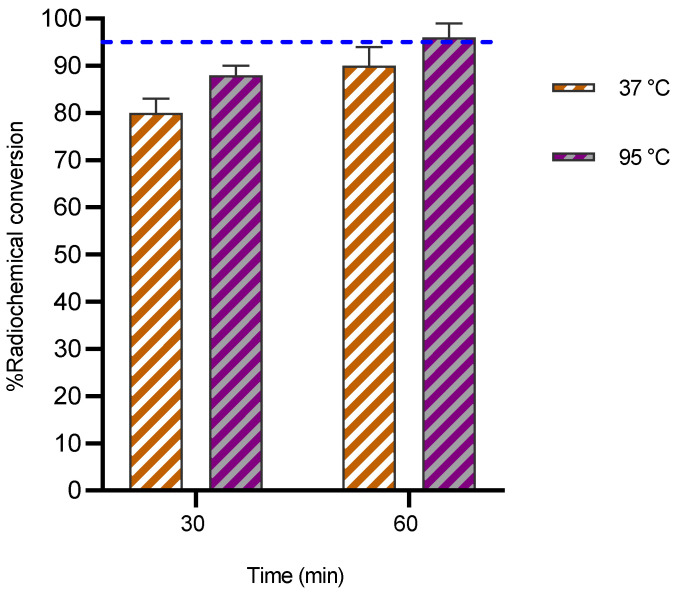
Radiochemical conversions obtained for 3p-*C*-NETA (10 µM) labeling with ^67^Cu (10 MBq; 0.15 M NH_4_OAc, pH 5.8) at 37 °C and 95 °C. Blue line is inserted to indicate a yield of 95%.

Because of its highly desirable decay characteristics (T_1/2_: 2.58 d, mean E_β_-: 141 keV, E_β_-_max_: 562 keV) 31, copper-67 is considered as a promising radionuclide for radioimmunotherapy. Currently, NOTA remains the chelator of choice for copper radionuclides as it labels efficiently at rt 32. To explore the binding kinetics of ^67^Cu with 3p-C-NETA, radiolabeling experiments were performed. At 37 °C, RCCs of 80 ± 1.9% and 88.3 ± 2.5% were observed at 30 and 60 min respectively with a ligand concentration of 10 µM at 37 °C (**Figure 4**). Increasing the temperature to 95 °C improved the RCCs to 90.4 ± 2.1% and 96.1 ± 3.2% at 30 and 60 min respectively.

#### ^213^Bi

**Figure 5:**
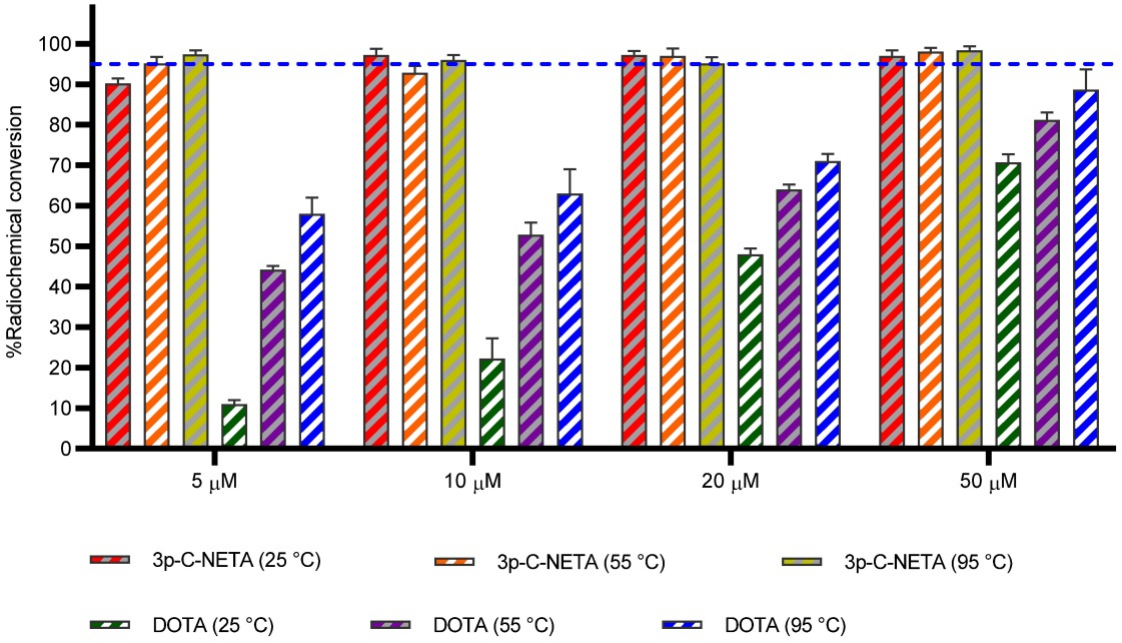
Radiochemical conversions obtained for 3p-C-NETA and DOTA with ^213^Bi. Ligand concentration of 5, 10, 20, 50 µM (0.37 M TRIS buffer, pH 8.5) with ^213^Bi (200-220 kBq, 5 min) at 25, 55 and 95 °C. Blue line is inserted to indicate a yield of 95%.

Kang *et al.* have reported the labeling of 3p-*C*-NETA with Bi(III), however, in this experiment the long-lived radionuclides ^205^Bi and ^206^Bi were used as surrogate for ^213^Bi 12. Here we report for the first time radiolabeling of 3p-*C*-NETA with the short-lived alpha-emitter ^213^Bi. Radiolabeling of 3p-*C*-NETA with ^213^Bi was extremely rapid and virtually completed in 5 min (90.3 ± 1.3%, 25 °C, 5µM; **Figure 5, Table S2**), compared to a lower complexation yield with DOTA (11.0 ± 1.0%) under the same reaction conditions. Maintaining the temperature at 25 °C and increasing the concentration of 3p-*C*-NETA to 10 µM and 20 µM resulted in a complexation yield of 95.2 ± 1.0% and 97.4 ± 1.0% respectively, whereas RCCs of only 22.3 ± 1.0 and 48.7 ± 4.0% were observed for DOTA. Only when using a concentration of 50 µM DOTA and a temperature of 95 °C, RCCs above 85% were observed (**Table S2**). In conclusion, a concentration of 10 µM 3p-*C*-NETA and a temperature of 25° is sufficient to obtain quantitative RCCs in only 5 min. This makes 3p-*C*-NETA an ideal chelator for ^213^Bi, as any type of heat sensitive biomolecule can be labeled using these mild conditions, and a kit-based labeling approach without final purification step might be possible 9. Advantageous in this context is that ^213^Bi can be easily supplied on site using a ^225^Ac/^213^Bi generator 33.

#### ^225^Ac

**Figure 6:**
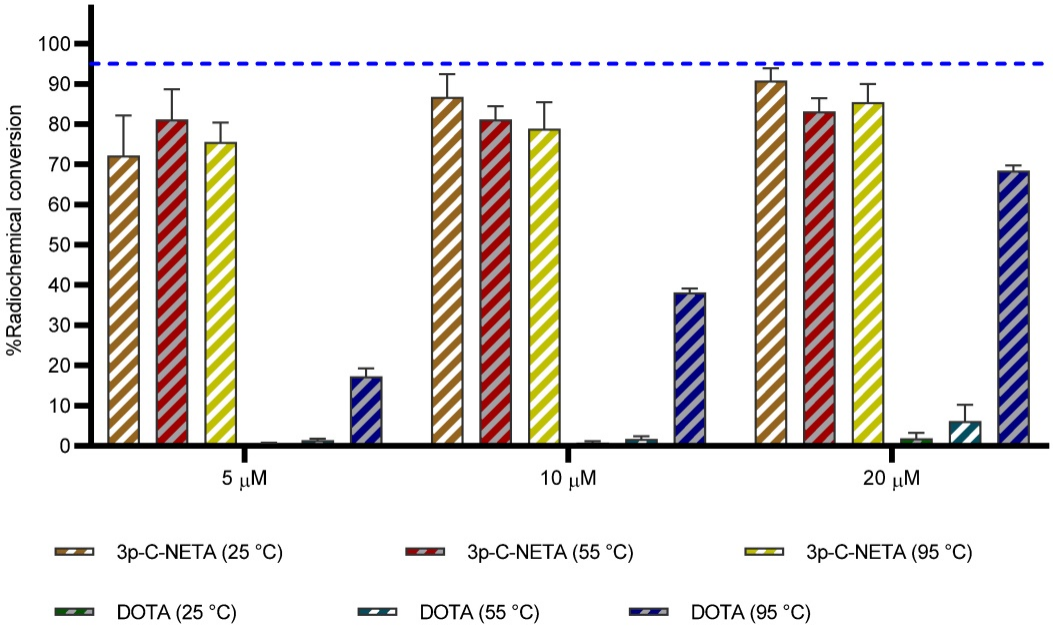
** Radiochemical conversions obtained for 3p-C-NETA and DOTA with ^225^Ac.** Ligand concentration of 5, 10, 20 µM (0.37 M TRIS buffer, pH 8.5) with ^225^Ac (90-100 kBq), 1 h, at 25, 55 and 95 °C. Blue line is inserted to indicate a yield of 95%.

The alpha emitter ^225^Ac has drawn particular attention in nuclear medicine due to the excellent decay properties (T_1/2_: 9.9 d, E_α_ = 5.8 MeV) it possesses. Promising results have been obtained with *e.g.* [^225^Ac]Ac-PSMA-617 for treatment of prostate cancer patients 34. DOTA can be used as chelator for ^225^Ac, but high temperatures (80-95 °C, microwave) are required to obtain good radiochemical conversions. Hence, the goal of this experiment was to determine the complexation potential of 3p-*C*-NETA with ^225^Ac using DOTA as a benchmark. ^225^Ac complexed moderately to 3p-*C*-NETA (RCC of 72.2 ± 10% at 25 °C) at a concentration of 5 µM in 1 h, in contrast to DOTA that performed poorly using the same labeling conditions (RCC of 0.6 ± 0.2%). The RCC improved at a ligand concentration of 10 µM (RCC of 86.8 ± 5.6%, 25 °C) in 1 h, while RCC of less than 10% was observed for DOTA using the same reaction conditions. Surprisingly, increasing the temperature to 95 °C yielded no significant change in RCC for 3p-*C*-NETA, however, an improved RCC of 38.1 ± 1.0% and 68.4 ± 1.3% was observed for DOTA using a concentration of 10 µM and 20 µM respectively (**Figure 6, Table S3**).

### *In vitro* stability of 3p-*C*-NETA radiocomplexes in PBS and human serum

**Figure 7:**
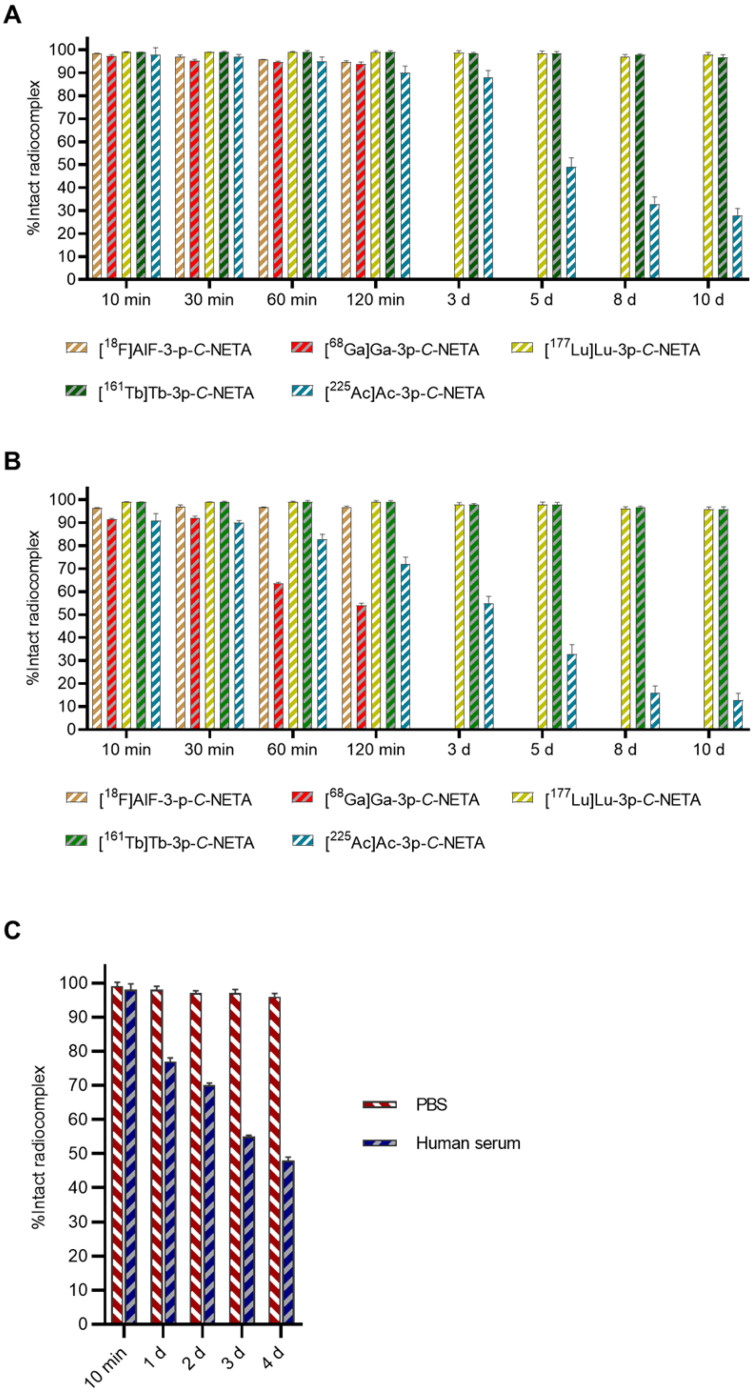
*In vitro* stability studies in** (A)** PBS and** (B)** human serum. Short-lived radiocomplexes ([^18^F]AlF-3-p-C-NETA and [^68^Ga]Ga-3p-C-NETA) were studied up to 240 min, however, studies up to 120 min are shown as there was no significant difference, while long-lived radiocomplexes ([^177^Lu]Lu-3p-C-NETA, [^161^Tb]Tb-3p-C-NETA and [^225^Ac]Ac-3p-C-NETA) were studied up to 10 d; **(C)** *In vitro* stability studies in PBS and human serum for [^67^Cu]Cu-3p-C-NETA.

Based on the rapid radiolabeling kinetics of 3p-*C*-NETA, we decided to test the stability of the different radiocomplexes *in vitro* (**Figure 7**). The Al^18^F-labeled 3p-*C*-NETA complex showed excellent stability as 94.4 ± 0.3% and 92.4 ± 1.2% of intact radiocomplex was observed at 240 min in PBS and in human serum, respectively. The ^68^Ga-complex was stable in PBS (>90%, 2 h), but ^68^Ga dissociated rapidly in human serum as only 63.7 ± 0.4% of the intact radiocomplex was observed at 60 min.

As expected, and reported before, >95% of the ^177^Lu and ^161^Tb-3p-*C*-NETA complex remained intact in PBS and human serum, even after 10 d incubation. Surprisingly, the ^67^Cu-complex showed excellent stability in PBS (>95% intact complex after 4 d), but only ~80% intact radiocomplex was observed in human serum after 24 h and less than 50% after 4 d incubation. The ^225^Ac-labeled complex was not stable as pronounced dissociation was observed both in PBS (<40% intact complex after 10 d incubation) and human serum (<20% intact complex after 10 d incubation). This observation might be explained by the relatively big ionic radius of ^225^Ac making it harder for 3p-*C*-NETA to encapsulate Ac^3+^ into its coordination sphere. The *in vitro* radiocomplex stability data indicate that 3p-*C*-NETA is a promising ligand for the diagnostic Al^18^F, and therapeutic radionuclides ^177^Lu and ^161^Tb. Moreover, the excellent stability of the Bi-3p-*C*-NETA complex was reported before 35. Based on this data, it is clear that 3p-*C*-NETA is not suited as ligand for ^68^Ga, ^67^Cu and ^225^Ac.

### 3p-*C*-NETA-TATE metal complex membrane-based affinity studies

To illustrate the versatility of the 3p-*C*-NETA chelator, we conjugated it to the somatostatin analogue (Tyr^3^)-octreotate leading to 3p-*C*-NETA-TATE. This allows radiosynthesis, using the same precursor molecule, of both diagnostic and therapeutic radiopharmaceuticals for NET imaging and treatment, respectively. We assessed the *in vitro* binding characteristics (IC_50_) of [^nat^Tb]Tb-3p-*C*-NETA-TATE, [^nat^Bi]Bi-3p-*C*-NETA-TATE, [^nat^Lu]Lu-3p-*C*-NETA-TATE and [^nat^F]AlF-3p-*C*-NETA-TATE on SSTR2 overexpressing CHO-K1 cell membranes using [^111^In]In-DOTATATE as competing ligand. SSTR2 overexpressing cell membranes were used in this assay as in the majority of NETs, SSTR2 is the most abundant SSTR, even when other subtypes are present 2. The IC_50_ values of DOTATATE and [^nat^F]AlF-NOTA-Octreotide were also determined for comparison. Small structural changes in the vector molecule, chelator substitution, or metal replacement had an effect on binding characteristics (**Table 1**). Excellent binding for SSTR2 was found for [^nat^Lu]Lu-3p-*C*-NETA-TATE (IC_50_ 15.4 ± 5.3 nM) and [^nat^F]AlF-3p-*C*-NETA-TATE (IC_50_ 19.0 ± 6.0 nM) with slightly lower IC50 values compared to the reference compound [^nat^F]AlF-NOTA-Octreotide (IC_50_ 25.7 ± 7.9 nM). The IC_50_ value for [^nat^Tb]Tb-3p-*C*-NETA-TATE (IC_50_ 27.0 ± 5.0 nM) was comparable with the reference compound. However, the IC_50_ value for [^nat^Bi]Bi-3p-*C*-NETA-TATE (IC_50_ 56.0 ± 21.4 nM) was 2-fold higher compared to [^nat^F]AlF-NOTA-Octreotide. However, this is still still in the nanomolar range, which should be sufficient for efficient NET targeting in an *in vivo* setting where SSTR2 is overexpressed. Interestingly, the metal free DOTATATE demonstrated the lowest IC_50_ value (IC_50_ 4.6 ± 2.1 nM). Reubi *et al.* previously suggested that these noticeable changes in receptor affinity profiles are not only due to the different charges but also to the different chemical structures and hydrophilicity of these compounds 36.

### Synthesis and stability studies of [^18^F]AlF-3p-*C*-NETA-TATE

**Figure 8:**
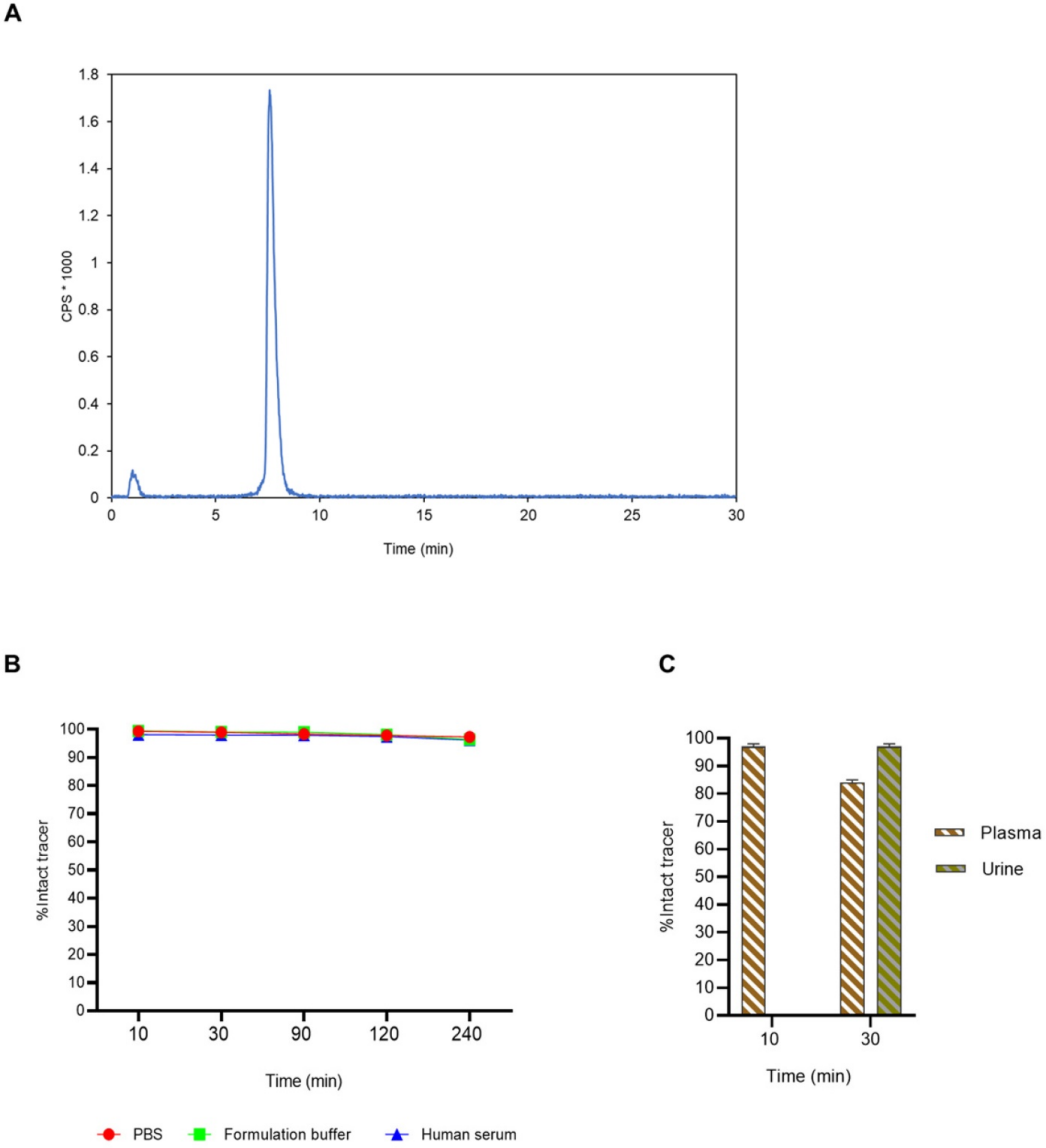
** (A)** HPLC radiochromatogram of [^18^F]AlF-3p-C-NETA-TATE after automated radiosynthesis and purification. A RCP of 96.8% ([^18^F]AlF-3p-C-NETA-TATE, rt 7-9 min) was observed with 3.2% free [^18^F]F^-^ or [^18^F]AlF (rt: 0.5-1 min). **(B)** *In vitro* stability of [^18^F]AlF-3p-C-NETA-TATE. The stability of [^18^F]AlF-3p-C-NETA-TATE was evaluated in PBS, formulation buffer and human serum at 37 °C using radioHPLC. **(C)** *In vivo* radiometabolite study of [^18^F]AlF-3p-C-NETA-TATE in rats. Radiometabolites were quantified using radioHPLC and gammacounting in plasma (10 min p.i.) and urine (10 and 30 min p.i.) (±SD, n = 3).

3p-*C*-NETA-TATE was radiolabeled with [^18^F]AlF in an automated AllinOne® synthesis module ([^18^F]AlF-NOTA-octreotide method) 22 and the corresponding [^18^F]AlF-3p-*C*-NETA-TATE was obtained in good RCY [(41.4 ± 8%, (decay-corrected, activity final batch of purified product/activity in reactor, *n* = 8, apparent molar activity 22 ± 8 GBq/μmol)] and an excellent radiochemical purity ( >97%, **Figure 8A**). Using identical radiolabeling conditions, the RYC was even better than for [^18^F]AlF-NOTA-octreotide (RCY of 26.3 ± 3.6%) 37, illustrating the robustness of the developed automated Al^18^F-radiolabeling protocol and indicating that 3p-C-NETA is indeed a promising chelator for the Al^18^F-method. As a note, the starting activity of [^18^F]F^-^ was relatively low for the synthesis of [^18^F]AlF-3p-C-NETA-TATE used for these preclinical studies, resulting in a moderate apparent molar activity of 22 GBq/μmol which might have an effect on biodistribution and uptake in SSTR2-expressing organs. The same was observed for [^18^F]AlF-NOTA-octreotide (25 ± 7 GBq/μmol), but for clinical productions the apparent molar activity of [^18^F]AlF-NOTA-octreotide was 160 GBq/μmol by increasing the starting activity of [^18^F]F^-^. Therefore, similar increase of apparent molar activity for [^18^F]AlF-3p-C-NETA-TATE is also expected for future clinical productions.

Interestingly, despite the chiral center in the chemical structure of 3p-*C*-NETA, [^18^F]AlF-3p-*C*-NETA-TATE eluted as a single peak on the radioHPLC system and no formation of stereoisomers was observed. In contrast, radioHPLC of [^18^F]AlF-NOTA-octreotide showed the formation of two stereoisomers and the identity of both peaks was confirmed using radio-LC/HRMS analysis 22.

[^18^F]AlF-3p-*C*-NETA-TATE displayed excellent *in vitro* stability with >95% intact complex after 4 h in all tested conditions (human serum, PBS and formulation buffer, **Figure 8B**) and no radiolysis was observed.

### Radiometabolite studies [^18^F]AlF-3p-*C*-NETA-TATE

The *in vivo* metabolic stability of [^18^F]AlF-3p-*C*-NETA-TATE was studied in rats by analyzing plasma (10 and 30 min post-injection) and urine (30 min post-injection) using radioHPLC. More than 97% of intact [^18^F]AlF-3p-*C*-NETA-TATE was present in plasma (10 min p.i.) while at 30 min p.i., 84 ± 10.2% of intact [^18^F]AlF-3p-*C*-NETA-TATE was observed compared to >98% of [^18^F]AlF-NOTA-Octreotide. This indicates slightly lower *in vivo* plasma stability of the [^18^F]AlF-3p-*C*-NETA complex compared to the [^18^F]AlF-NOTA-complex **(Figure S1)** 22. At 30 min p.i. in urine, >97% of intact [^18^F]AlF-3p-*C*-NETA-TATE was observed which was comparable as reported for [^18^F]AlF-NOTA-Octreotide (**Figure 8C**) 22.

### *In vivo* biodistribution of [^18^F]AlF-3p-*C*-NETA-TATE

**Figure 9:**
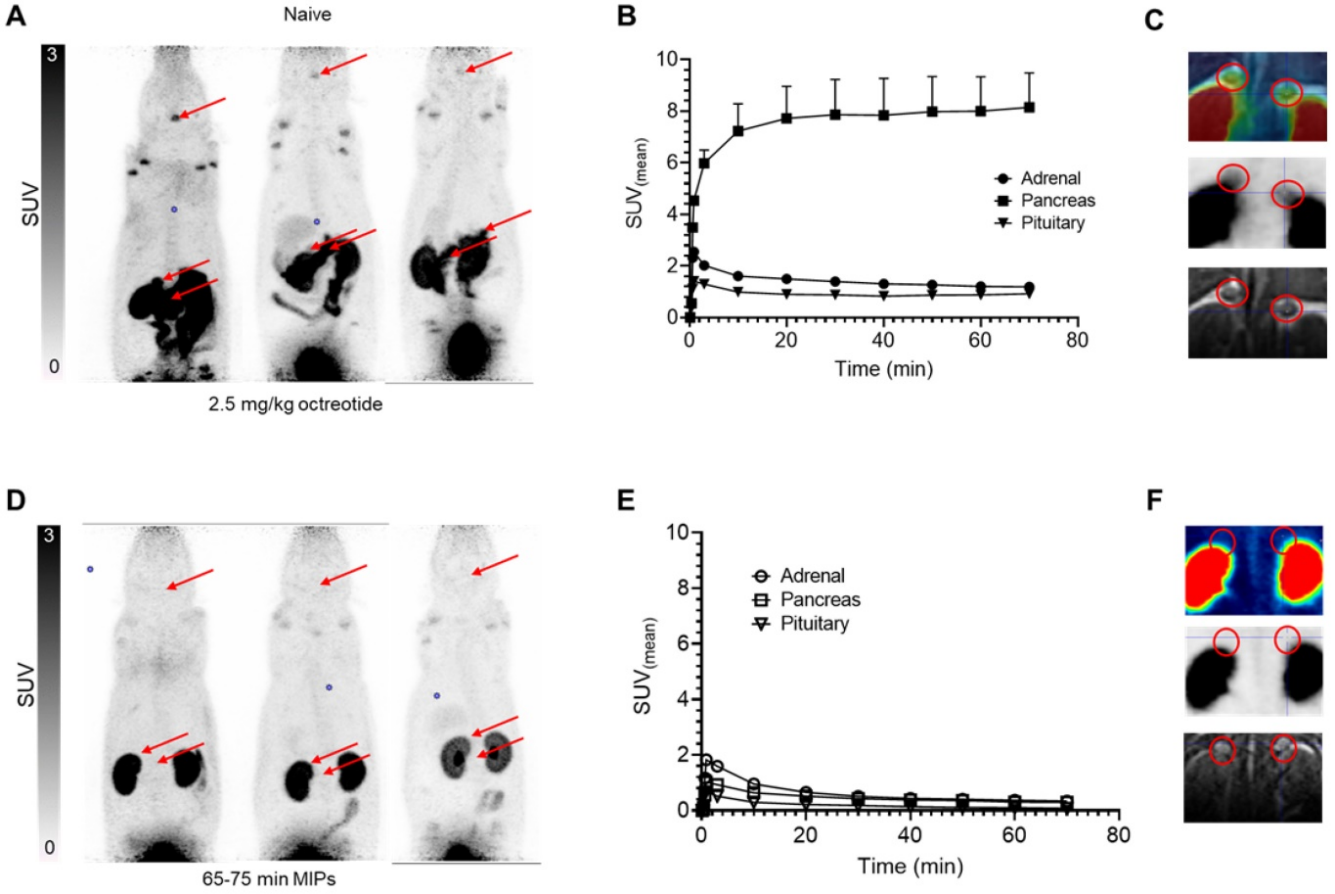
***In vivo* biodistribution of [^18^F]AlF-3p-C-NETA-TATE in rats using control and blocking conditions (2.5mg/kg octreotide). A,D)** Maximum intensity projections (PET) at 65-75 minutes post-injection, with SSTR2-expressing organs (pituitary and adrenal glands, pancreas) highlighted by arrows in representative naïve (top) and blocked (bottom) animals. **B,E)** Time activity curves (TACs) of SSTR2-expressing organs in naïve (top) and blocked (bottom) animals (n=3), demonstrating high and blockable SSTR2 uptake. **C,F)** Coronal slices indicating delineation of adrenal glands on MRI sequence subsequently applied to PET data to generate TAC. (upper): PET-MRI fusion, (middle): PET (lower): MRI (PET images from 65-75 min timepoint).

The µPET/MR studies in normal rats demonstrated fast clearance from blood and high uptake of [^18^F]AlF-3p-*C*-NETA-TATE in SSTR2-expressing organs (**Figure 9),** which was significantly blocked upon a co-injection of 2.5 mg/kg octreotide acetate (75 min p.i.: adrenals SUV 1.20 ± 0.59 vs 0.24 ± 0.03, pituitary SUV 0.93 ± 0.34 vs 0.20 ± 0.04 and pancreas SUV 7.78 ± 4.26 vs 0.34 ± 0.20, all p<0.05), indicating SSTR2 specific uptake. The tracer concentration in blood and non-SSTR2 expressing organs was low (SUV<0.6) at 2 h post-injection (adrenals SUV 1.22 ± 0.65 vs 0.18 ± 0.04, pituitary SUV 0.93 ± 0.36 vs 0.21 ± 0.06, pancreas SUV 8.35 ± 5.07 vs 0.31 ± 0.07), resulting in high contrast images. [^18^F]AlF-3p-*C*-NETA-TATE thus demonstrated excellent uptake in SSTR2 expressing organs (adrenals, pancreas and pituitary gland), with comparable or higher uptake than seen with [^18^F]AlF-NOTA-Octreotide 22. As observed with [^18^F]AlF-NOTA-Octreotide, moderate bone uptake was observed for [^18^F]AlF-3p-*C*-NETA but did not increase in function of time (**Figure S2**), indicating only minor defluorination and high *in vivo* stability. Further, whilst the *in vivo* plasma radiometabolite study indicated a slightly lower stability for the [^18^F]AlF-3p-*C*-NETA complex compared to the [^18^F]AlF-NOTA-complex, bone uptake observed in the spine at 2 h p.i. in naïve rats was comparable for both [^18^F]AlF-3p-*C*-NETA-TATE (SUV 0.43 ± 0.25) and [^18^F]AlF-NOTA-octreotide (SUV 0.45 ± 0.03). As [^18^F]AlF-NOTA-octreotide has been used already in a clinical setting without disturbing bone signal, it is not expected that this minor defluorination would complicate clinical translation of the 3p-*C*-NETA chelator 6. Further, defluorination (decomplexation of [^18^F]AlF) and resulting bone uptake might be more pronounced in rodents than in humans, as is also observed for ^89^Zr-labeled radiopharmaceuticals (decomplexation of ^89^Zr in this case) 38. Preclinical *in vivo* studies in tumor-bearing mice are needed to assess the *in vivo* stability further. These promising* in vitro* and *in vivo* results for the theranostic 3p-*C*-NETA ligand supports further preclinical and potentially, in a later phase, clinical studies.

## Conclusion

3p-*C*-NETA is an excellent and versatile chelator that can be used for both targeted radionuclide therapy (^177^Lu, ^213^Bi, ^161^Tb) and diagnostic applications using the well-established Al^18^F-method. Further, as proof of concept, [^18^F]AlF-3p-*C*-NETA-TATE was obtained in good RCY and excellent radiochemical purity and showed promising pharmacokinetic properties. [^213^Bi]Bi-3p-*C*-NETA-TATE and [^177^Lu]Lu-3p-*C*-NETA-TATE will be evaluated as potential therapeutic partners for NETs in a theranostic setting in combination with [^18^F]AlF-3p-*C*-NETA-TATE as the diagnostic partner using SSTR2 overexpressing mouse tumor models. 3p-*C*-NETA has the potential to significantly expand the theranostic radiochemical space for nuclear medicine applications.

## Supplementary Material

Supplementary methods, figures and tables.

## Figures and Tables

**Table 1:** IC_50_ values of the 3p-C-NETA-TATE metal complexes and reference compounds using SSTR2 overexpressing CHO-K1 cell membranes (±SD, n = 3 (independent biological assays))

Compound	IC_50_ (nM)
[^nat^Tb]Tb-3p-*C*-NETA-TATE	27.0 ± 5.0
[^nat^Bi]Bi-3p-*C*-NETA-TATE	56.0 ± 21.4
[^nat^Lu]Lu-3p-*C*-NETA-TATE	15.4 ± 5.3
[^nat^F]AlF-3p-*C*-NETA-TATE	19.0 ± 6.0
[^nat^F]AlF-NOTA-Octreotide	25.7 ± 7.9
DOTATATE	4.6 ± 2.1
