# Supplementary material for: 3p-C-NETA: A versatile and effective chelator for development of Al18F-labeled and therapeutic radiopharmaceuticals
Source: Theranostics. 2022 Aug 8;12(13):5971–85. doi: 10.7150/thno.75336 (PMC9373814; doi:10.7150/thno.75336)
Supplement: Supplementary file 1 — Supplementary methods, figures and tables. [file thnov12p5971s1.pdf]

3p-*C*-NETA: A Versatile and Effective Chelator for development of Al^{18}F-labeled and Therapeutic Radiopharmaceuticals

Stephen Ahenkorah[1,2], Erika Murce[3], Christopher Cawthorne[4], Jessica Pougoue Ketchemen[5], Christophe M. Deroose[4], Thomas Cardinaels[1,6], Yann Seimbille[3,7], Humphrey Fonge[5,8], Willy Gsell[9], Guy Bormans[2], Maarten Ooms[1,] and Frederik Cleeren[2,]**

[1] NURA, Belgian Nuclear Research Center (SCK CEN), Mol, Belgium.

[2] Radiopharmaceutical Research, Department of Pharmaceutical and Pharmacological sciences, University of Leuven, Leuven, Belgium.

[3] Department of Radiology and Nuclear Medicine, Erasmus MC, Rotterdam, the Netherlands.

[4] Nuclear Medicine and Molecular Imaging, Department of Imaging and Pathology, University of Leuven, Leuven, Belgium.

[5] Department of Medical Imaging, College of Medicine, University of Saskatchewan, Saskatoon, Canada.

[6] Department of Chemistry, University of Leuven, Leuven, Belgium.

[7] Life Sciences Division, TRIUMF, Vancouver, Canada.

[8] Department of Medical Imaging, Royal University Hospital (RUH), Saskatoon, Canada.

[9] Biomedical MRI/MoSAIC, Department of Imaging and Pathology, Biomedical Sciences Group, KU Leuven, Leuven, Belgium

*Correspondence: maarten.ooms@sckcen.be (M.O) and frederik.cleeren@kuleuven.be (F.C)

Phone: +32 14333283 (M.O) and +32 16377237 (F.C)

Instrumentations

3p-*C*-NETA: ^{1}H, and ^{13}C NMR spectra were obtained using a Bruker 300 instrument (Billerica, MA, USA). Chemical shifts were reported in parts per million (ppm) on the δ scale using residual protio-solvent signals (^{1}H NMR, $CDCl_3$ δ 7.26, DMSO-d6 δ 2.50, MeOH-d_4 δ 3.31; ^{13}C NMR, $CDCl_3$ δ 77.0, DMSO-d_6 δ 9.5, MeOH-d_4 δ 49.0) as internal references. Reactions were monitored by thin layer chromatography (TLC) using pre-coated aluminium plates (silica gel 60, F254, Merck). Nominal mass spectra of the intermediates and accurate mass of the final compounds were determined by a Dionex Ultimate 3000 LC System (Dionex, Germering, Germany) hyphenated to a high resolution time-of-flight mass spectrometer (maXis impact, Bruker, Bremen, Germany) equipped with an orthogonal electrospray ionization interface (UPLC-HRMS). Acquisition and processing of data were performed using HyStar® and Compass DataAnalysis® (version 4.1, Bruker) respectively. Spectra were obtained in either positive or negative ionization mode based on the compound type. Refer to Kang *et al*., 2015 for the synthesis of compound **1**. Melting point (mp) of compounds **1**, **2** and **3** were determined by Krüss Melting Point Meter M5000 (Hamburg, Germany)3p-*C*-NETA-TATE: Liquid phase reactions were monitored by LC-MS (Agilent 1260 Infinity II Infinity Lab LC/MSD XT system) using an Infinity Lab Poroshell 120 EC-C_{18} column (3 x 100 mm, 2.7 μm). The mobile phase consisted of: solvent A, 0.1% formic acid in water; solvent B, 0.1% formic acid in acetonitrile. The following LC gradient was used for all analyses: 0-5 min, 5-100% B and 5-8 min, 100% B at a flow rate of 0.5 mL/min. Purification was performed by preparative HPLC using an Agilent 5 Prep C18 column (50 x 21,2 mm) and the following gradient:

0-8 min, 5-100% B; 8-10 min, 100% B at a flow rate of 10 mL/min (solvent A: 0.1% formic acid in water; solvent B: 0.1% formic acid in acetonitrile).

Synthesis of 3p-*C*-NETA-oxa-(*t*Bu)-butanoic acid and 3p-*C*-NETA-TATE

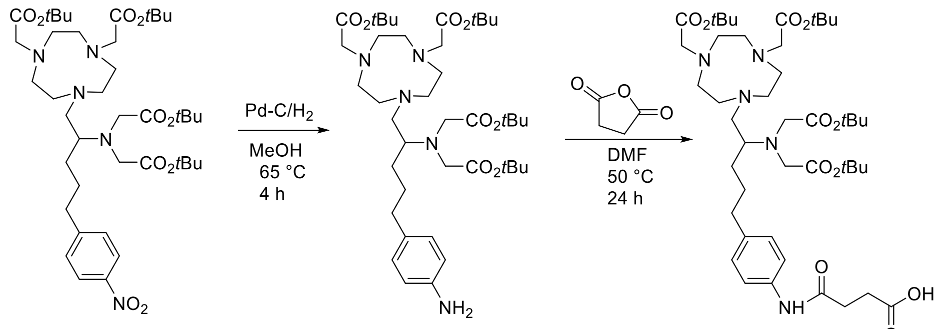

1 2 (95%) 3 (92%)

Scheme S1. synthetic route for 3p-C-NETA-(tBu)-oxa-butanoic acid

di-tert-Butyl 2,2'-((1-(4,7-bis(2-(tert-butoxy)-2-oxoethyl)-1,4,7-triazonan-1-yl)-5-(4-nitrophenyl)pentan-2-yl)azanediyl)diacetate (1)

Yield: 82.4%; mp: 55.4-55.7 °C; ^{1}H NMR (300 MHz, MeOD) δ 8.19 (d, *J* = 8.7 Hz, 2H), 7.48 (d, *J* = 8.7 Hz, 2H), 3.54 (d, *J* = 4.9 Hz, 4H), 3.49 (s, 2H), 3.41 (s, 2H), 3.33 (dt, *J* = 3.3, 1.6 Hz, 15H), 3.25 (d, *J* = 1.3 Hz, 1H), 3.19 (d, *J* = 4.7 Hz, 1H), 3.16 (s, 2H), 1.73 (dd, *J* = 8.1, 4.2 Hz, 2H), 1.56 – 1.43 (m, 36H); ^{13}C NMR (300 MHz, MeOD) δ 175.4, 173.6, 172.6, 171.8, 133.4, 131.8, 128.1, 128.7, 114.6, 117.3, 82.3, 81.1, 80.41, 58.3, 57.9, 57.4, 54.8, 54.7, 53.3, 51.8, 51.5, 47.5, 47.7, 47.3, 44.6, 35.1, 28.1 (14C), 27.7; High resolution mass spectrometry (HRMS: ESI-MS): $C_{41}H_{69}N_5O_{10}$ [M + H]$^+$ m/z 792.5112, Found [M + H]$^+$ m/z 792.5113.

Synthesis and characterization of di-*tert*-Butyl 2,2'-((5-(4-aminophenyl)-1-(4,7-bis(2-(tert-butoxy)-2-oxoethyl)-1,4,7-triazonan-1-yl)pentan-2-yl)azanediyl)diacetate (2)

To a solution of **1** (80 mg, 0.10 mmol) in MeOH (10 mL) at 65 °C was added dry 10% Pd/C (4 mg) under argon. The reaction mixture was placed under hydrogenation apparatus for 4 h. The resulting mixture was filtered via celite bed and washed thoroughly with MeOH. The filtrate was concentrated in vacuo to provide **2** (46 mg, 95%**;** mp: 54.6-55.3°C); ^{1}H NMR (300 MHz, MeOD) δ 6.94 (d, *J* = 8.2 Hz, 2H), 6.66 (d, *J* = 8.2 Hz, 2H), 4.84 (s, 10H), 3.49 – 3.43 (m, 4H), 3.30 (s, 4H), 3.13 (d, *J* = 11.5 Hz, 4H), 2.77 (s, 4H), 2.53 (d, *J* = 6.9 Hz, 2H), 1.47 (d, *J* = 3.7 Hz, 39H); ^{13}C NMR (300 MHz, MeOD) δ 172.6, 171.3, 171.6, 170.9, 131.7, 131.0, 129.1, 128.3, 115.9, 115.1, 81.8, 81.4, 80.61, 59.3, 58.11, 57.8, 55.6, 55.1, 53.4, 52.9, 51.3, 48.9, 48.4, 47.4, 46.1, 34.7, 28.3, 27.0 (14C); HRMS(ESI-MS): $C_{41}H_{71}N_5O_8$ $[M + H]^+$ m/z 762.5659, Found $[M + H]^+$ m/z 762.5658.

Synthesis and characterization of 4-((4-(5-(4,7-bis(2-(tert-butoxy)-2-oxoethyl)-1,4,7-triazonan-1-yl)-4-(bis(2-(tert-butoxy)-2-oxoethyl)amino)pentyl)phenyl)amino)-4-oxobutanoic acid (3)

To a solution of **2** (45 mg, 1.0 eq, 0.06 mmol) in DMF (5 mL) was added succinic anhydride (8.9 mg, 1.5 equiv., 0.088 mmol). The resulting mixture was stirred at 50 °C for 24 h followed by concentration in vacuo. The crude mixture was washed with 1% acetic acid followed by HPLC purification to provide **3** (46.2 mg, 92%; mp: 57.1-57.8 °C); ^{1}H NMR (300 MHz, $CDCl_3$) δ 10.06 (s, 1H), 7.58 (d, *J* = 7.8 Hz, 2H), 7.04 (d, *J* = 8.9 Hz, 2H), 3.70 (s, 4H), 3.52 – 3.35 (m, 14H), 2.96 (s, 4H), 2.89 (s, 2H), 2.67 (dd, *J* = 10.8, 4.3 Hz, 16H), 2.10 (s, 2H), 1.51 (d, *J* = 1.7 Hz, 4H), 1.38 (d, *J* = 1.3 Hz, 6H), 1.25 (s, 4H), 0.86 (d, *J* = 7.2 Hz, 2H), 0.06 (s, 1H), 0.01 (s, 10H), 0.07 (s, 1H); ^{13}C NMR (300 MHz, MeOD) δ 175.0 (4C), 171.7 (2C), 137.4, 136.6, 128.4, 120.0, 81.6 (2C), 81.1(2C), 58.2, 57.8, 55.3, 54.2, 52.7, 51.1, 50.4, 48.4, 48.2, 47.0, 46.7, 34.8 (2C), 31.0, 29.4 (2C),

28.6 (2C), 28.2, 27.3 (12C). HRMS (ESI-MS): $C_{45}H_{75}N_5O_{11}$ $[M + H]^+$ m/z 862.5507, Found $[M + H]^+$ m/z 862.5505 ± 0.02Da.

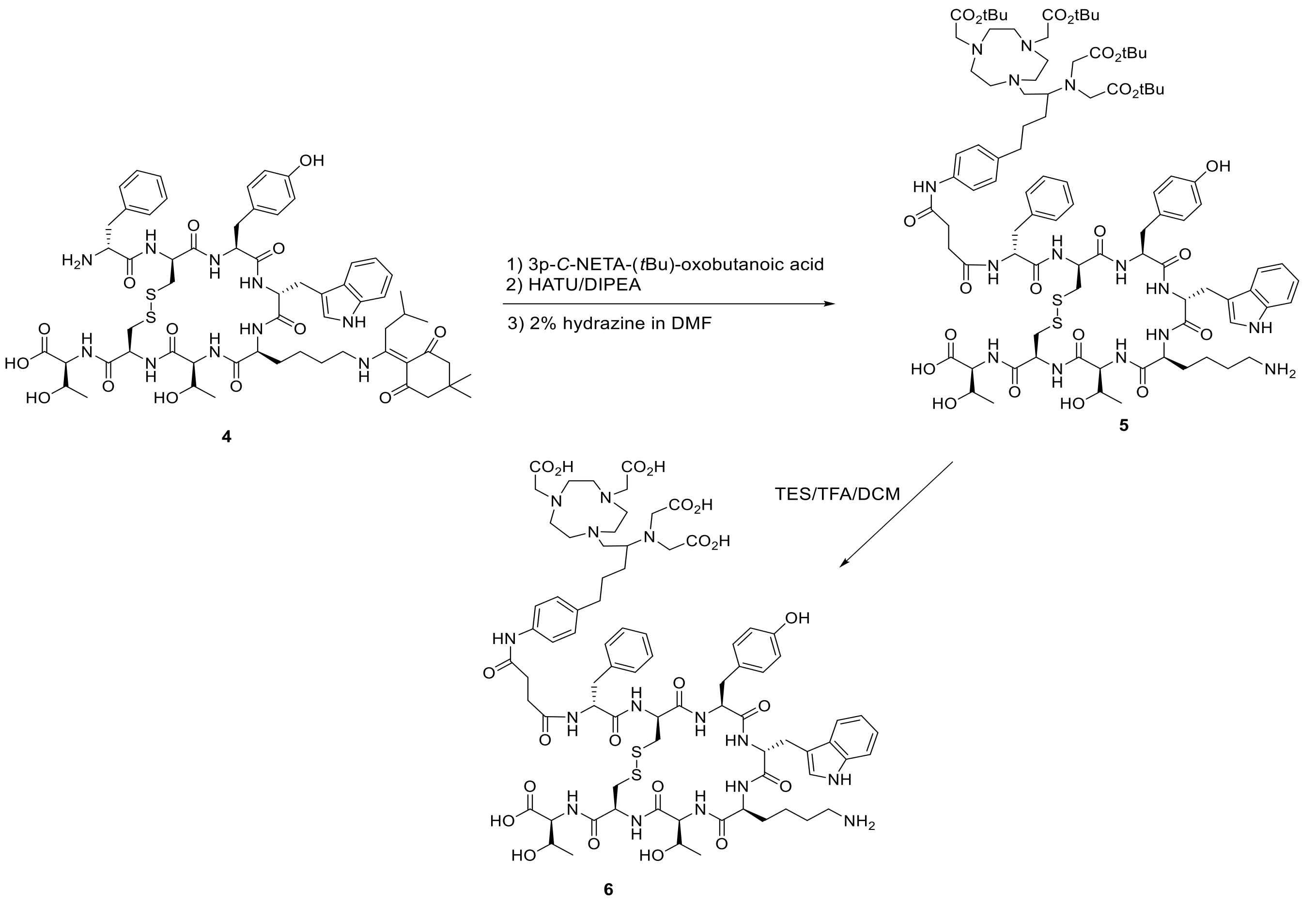

Scheme S2. Synthetic route for 3p-*C*-NETA-TATE

Synthesis of D-Phe-cyclo[Cys-Tyr-D-Trp-Lys(IvDde)-Thr-Cys]-Thr-OH (4) The protected peptide D-Phe-Cys(Acm)-Tyr(*t*Bu)-D-Trp(Boc)-Lys(ivDde)-Thr(*t*Bu)-Cys(Acm)-Thr(*t*Bu)-OH was synthesized by standard Fmoc solid-phase peptide synthesis protocols. Conjugation of the Fmoc protected amino acids (4.0 equiv.) to the solid support was carried out in dimethylformamide

(DMF) using hexafluorophosphate azabenzotriazole tetramethyl uronium (HATU) (3.8 equiv.) and *N*,*N*-diisopropylethylamine (DIPEA) (7.8 equiv.) for 45 min. Fmoc deprotection was accomplished by treatment of the resin with a 20% solution of 4-methylpiperidine in DMF. Amide formation and Fmoc deprotection were monitored by Kaiser test. Coupling or Fmoc deprotection were performed twice when the reaction was not completed. Peptide synthesis was started by loading Fmoc-L-Thr(*t*Bu)-OH onto the 2-chlorotrityl chloride resin (0.25 g, loading capacity: 1.6 mmol/g). The resin was agitated for 90 min at 25 °C. The resin was capped using 8 mL of DCM/MeOH/DIPEA (v/v/v = 80:15:5) for 15 min at 25 °C. Subsequent Fmoc deprotection and coupling with Fmoc-L-Cys(Acm)-OH, Fmoc-L-Thr(*t*Bu)-OH, Fmoc-L-Lys(ivDde)-OH, Fmoc-D-Trp(Boc)-OH, Fmoc-L-Tyr(*t*Bu)-OH, Fmoc-L-Cys(Acm)-OH and Fmoc-D-Phe-OH were achieved following the same protocol described above. The protected linear peptide was then cyclized on resin by treatment with $Tl(TFA)_3$ (2.0 equiv.) in DMF. Completion of the cyclization was monitored by LC-MS. A solution of TFA/H_2O/TIPS (v/v/v = 95:2.5:2.5) was added to cleave the peptide from the solid support, and the mixture was stirred for 2 h at 25°C. The resin was removed from the solution by filtration, and ice-cold diethyl ether was added to the filtrate to precipitate the peptide. The crude peptide was purified by preparative HPLC to yield 200 mg of **4** (40%). ESI-MS: m/z calculated = 1255.54 $[M+H]^+$, found = 1255.54 $[M+H]^+$.

Synthesis of 3p-C-NETA(*t*Bu)$_4$-TATE (**5**)

30 mg of **4** (2.0 equiv.) were dissolved in 500 μL of DMF. 9 mg of 3p-C-NETA-(*t*Bu)-oxobutanoic acid (3, 10 μmol), 7.6 mg of 1-[bis(dimethylamino)methylene]-1H-1,2,3-triazolo[4,5-b]pyridinium 3-oxide hexafluoro phosphate (HATU, 2.0 equiv.) and 34.8 μL of DIPEA (2.0 equiv.) were added to the solution. The reaction was monitored by LC-MS. Upon completion, 15 μL of hydrazine

monohydrate were added to the solution (for a final concentration of 2% hydrazine in DMF). IvDde deprotection was followed by LC-MS and quantitative deprotection was obtained in 15 min. The reaction mixture was concentrated in vacuo and then purified by preparative HPLC to yield 7 mg (>95% purity) of **5** as a white solid (37%). ESI-MS: m/z calculated = 1893.33 $[M+H]^{+}$; found = 947.30 $[M+2H]^{2+}$ z=2·

Synthesis of 3p-*C*-NETA-TATE (6)

tert-butyl deprotection was performed by treatment of **5** with 1 mL of a solution of TFA/DCM/TES (v/v/v = 44:44:2) for 5 h. The mixture was concentrated under nitrogen flow and the product was purified by preparative HPLC to yield 3.5 mg (>95% purity) of **6** as a white solid (56%). ESI-MS: m/z calculated = 1668.90 $[M+H]^{+}$; found = 835.10 $[M+2H]^{2+}$ (z=2)

Table S1: Radiochemical conversion (%) of 3p-*C*-NETA with $Al^{18}F$, ^{68}Ga, ^{177}Lu and ^{161}Tb

**Temp/°C**	**$Al^{18}F$**	**^{68}Ga**	**^{177}Lu**	**^{161}Tb**
**25**	2.8 ± 0.2	68.7 ± 0.7	99.4 ± 0.4	68.9 ± 0.8
**40**	8.8 ± 0.4	87.9 ± 0.4	99.3 ± 0.1	78.2 ± 0.7
**55**	18.4 ± 0.7	91.7 ± 0.7	99.5 ± 0.7	96.7 ± 0.1
**95**	62.3 ± 1.5*	97.7 ± 0.1	99.6 ± 0.1	97.9 ± 0.1

***A RCC of 80.3 ± 0.6% was observed with a reaction content of 50% absolute EtOH (v/v)**

Table S2: Radiochemical conversion (%) of 3p-*C*-NETA and DOTA labeled with ^{213}Bi

Temp/°C	Conc/µM	3p-*C*-NETA				DOTA			
		5 min	10 min	15 min	20 min	5 min	10 min	15 min	20 min

25	5	90.3 ± 1.3	91.7 ± 0.9	90.8 ± 1.1	90.3 ± 1.0	11.0 ± 1.0	22.0 ± 1.3	28.0 ± 2.1	31.0 ± 2.3
	10	95.2 ± 1.0	94.5 ± 1.2	94.5 ± 0.7	95.3 ± 1.0	22.3 ± 1.0	64.5 ± 6.0	60.9 ± 1.7	55.3 ± 3.0
	20	97.4 ± 1.0	96.7 ± 0.8	96.5 ± 1.1	96.9 ± 1.0	48.7 ± 4.0	66.1 ± 3.1	62.5 ± 2.4	61.9 ± 1.4
	50	97.3 ± 0.8	97.5 ± 1.0	96.8 ± 1.1	96.9 ± 0.0	70.7 ± 5.0	78.0 ± 1.4	79.9 ± 1.0	77.1 ± 2.4
55	5	92.9 ± 1.2	94.3 ± 1.0	94.6 ± 0.8	95.4 ± 1.0	44.2 ± 3.1	53.3 ± 1.4	54.5 ± 2.2	61.2 ± 0.9
	10	96.0 ± 1.0	95.3 ± 1.2	95.7 ± 0.6	96.2 ± 1.0	52.9 ± 6.4	71.8 ± 5.3	70.6 ± 2.3	75.5 ± 0.4
	20	97.3 ± 0.0	97.4 ± 0.0	96.9 ± 0.4	97.1 ± 0.0	64.0 ± 0.8	76.7 ± 4.7	71.5 ± 2.8	71.9 ± 0.7
	50	97.0 ± 1.1	97.2 ± 1.0	97.0 ± 0.3	97.2 ± 0.0	81.2 ± 1.9	80.8 ± 6.1	76.2 ± 0.2	74.1 ± 6.2
95	5	95.2 ± 0.0	97.1 ± 0.7	96.4 ± 0.3	96.6 ± 0.0	58.0 ± 1.2	68.0 ± 4.2	67.0 ± 3.3	69.0 ± 1.4
	10	97.0 ± 1.0	97.6 ± 0.0	98.0 ± 0.1	97.7 ± 0.4	63.7 ± 0.2	70.6 ± 5.1	65.2 ± 3.1	70.2 ± 2.7
	20	98.1 ± 0.6	97.6 ± 0.2	98.6 ± 0.0	98.6 ± 0.2	71.5 ± 2.7	88.4 ± 6.2	89.2 ± 4.7	88.2 ± 0.9

	50	98.4 ± 0.1	97.4 ± 0.0	98.6 ± 1.0	98.5 ± 0.0	88.4 ± 3.7	93.1 ± 1.0	89.6 ± 0.8	86.3 ± 6.1

Table S3: Radiochemical conversion (%) of 3p-C-NETA and DOTA labelled with ^{225}Ac

**Temp/°C**	**Conc/µM**	**1 h**		**2h**	
		**3p-*C*-NETA**	**DOTA**	**3p-*C*-NETA**	**DOTA**
**25**	5	72.2 ± 10.0	0.6 ± 0.2	69.5 ± 15.3	1.8 ± 2.3
	10	86.8 ± 5.6	0.8 ± 0.4	82.1 ± 4.7	1.0 ± 0.5
	20	90.8 ± 3.1	1.9 ± 1.4	88.4 ± 5.2	1.6 ± 1.1
**55**	5	81.2 ± 7.5	1.4 ± 0.7	79.3 ± 17.4	5.4 ± 7.6
	10	83.2 ± 3.3	1.7 ± 0.7	91.5 ± 0.2	2.1 ± 0.3
	20	83.5 ± 12.5	6.2 ± 4.1	90.2 ± 4.5	6.8 ± 3.1
**95**	5	75.6 ± 4.8	17.3 ± 2.0	86.8 ± 6.4	34.7 ± 11.6
	10	78.9 ± 6.6	38.1 ± 1.0	92.0 ± 3.4	60.8 ± 2.0
	20	85.5 ± 4.5	68.4 ± 1.3	94.1 ± 0.4	89.4 ± 1.7

Cold synthesis of ([^{nat}Tb]Tb-3p-*C*-NETA-TATE, [^{nat}Bi]Bi-3p-*C*-NETA-TATE and [^{nat}Lu]Lu-3p-*C*-NETA-TATE: A solution containing $TbCl_3{\cdot}6H_2O$, $BiCl_3$ or $LuCl_3$ (2 mM in 1.2 M sodium acetate, pH 5.5 equiv.) and 3p-*C*-NETA-TATE (0.8 mg, 0.4 mM, 1 equiv.) in a total volume of 0.5 mL was prepared. The reaction mixture was allowed to react for 30 min at a temperature of 95 °C. Metal-free water (20 mL) was used to dilute the reaction mixture after cooling for 10 min and this was loaded onto an activated Sep-Pak Plus Light C_{18}. The vial was rinsed with 2 mL metal-free water and this was also loaded onto the activated Sep-Pak Plus Light C_{18}. The corresponding complex was eluted with 1 mL absolute ethanol into a vial. The ethanol was evaporated under vacuum and 1 mL of metal-free water and 0.5 mL of acetonitrile (LC-MS grade) was added. The mixture was sonicated for 5 min and filtered using a Captiva PTFE + GF 0.45 µm filter from Agilent.

Cold synthesis of [^{nat}F]AlF-3p-*C*-NETA-TATE and [^{nat}F]AlF-NOTA-Octreotide: The reaction was started by adding 490 µL $AlCl_3$ (5.1 mM in 0.1 M sodium acetate, pH 4.1, 10 equiv.) to 10 µL NaF (10 mg/mL in 0.1 M sodium acetate, pH 4.1, 10 equiv.) and let to stir for 5 min. Ethanol absolute was then added to the aluminum mixture and later added to 1 mg 3p-C-NETA-TATE or NOTA-Octreotide. The complexation was subsequently conducted at 95°C for 30 min. Metal-free water (2 mL) was used to dilute the reaction mixture after cooling for 10 min and this was loaded onto an activated Sep-Pak Plus Light C_{18}. The vial was rinsed with 2 mL metal-free water and this was also loaded onto the activated Sep-Pak Plus Light C_{18}. The corresponding complex was eluted with 1 mL absolute ethanol into a vial. The ethanol was evaporated under vacuum and 1 mL of metal-free water and 0.5 mL of acetonitrile (LC-MS grade) was added. The mixture was sonicated for 5 min and filtered using a Captiva PTFE + GF 0.45 µm filter from Agilent. Table 4 summarizes the mass data and purity obtained for the cold synthesis.

Table S4: Summary of the mass data and purity obtained for the cold synthesis

**Complex**	**Theoretical neutral average mass (± 0.02Da) +ESI-LCMS**		**Purity (%)**
	Expected	Observed	
**([^{nat}Tb]Tb-3p-*C*-NETA-TATE**	1825.6405	1825.6407	>95
**[^{nat}Bi]Bi-3p-*C*-NETA-TATE**	1875.6955	1875.6956	>96
**[^{nat}Lu]Lu-3p-*C*-NETA-TATE**	1841.6588	1841.6590	>96
**[^{nat}F]AlF-NOTA-Octreotide**	1348.7545	1348.7547	>96

**[natF]AlF-3p-*C*-NETA-TATE**	1713.6951	1713.6950	>96

3p-*C*-NETA-TATE metal complex membrane-based affinity studies protocol

Prior to the assay, the plate filters were pre-soaked in 200 μL 0.1% polyethylenimine for 1 h at room temperature. The assay buffer consists of 25 mM HEPES pH 7.4, 10 mM $MgCl_2$, 1 mM $CaCl_2$ and 0.5% Bovine Serum Albumin (BSA), while the washing buffer consists of 50 mM Tris-HCl pH 7.4, 0.2% BSA. Human SSTR2+ membranes derived from CHO-K1 cells (1x 400 units, 25 μg protein/unit)were prepared as follows: 0.125 mL of membrane + 4.875 mL assay buffer (1:40 dilution). Following pre-incubation, the diluted membranes (178 μL), [^{111}In]In-DOTATATE (2 μL, 10^{-9} M), and various concentrations of the competing non-radioactive compounds (20 μL, 10^{-12}-10^{-5} M) were added to each well, for a total volume of 200 μl/well . The plates were incubated at rt for 90 min. After incubation, the solution in each well was filtered by using a vacuum manifold and followed by 10 washes with ice-cold wash buffer (200 μL, each wash was filtered through). Each plate filter was removed carefully, placed in a counting tube and activity was measured in the gamma counter. The results were analyzed with GraphPad Prism using a binding competitive one site-fit log IC_{50} algorithm.

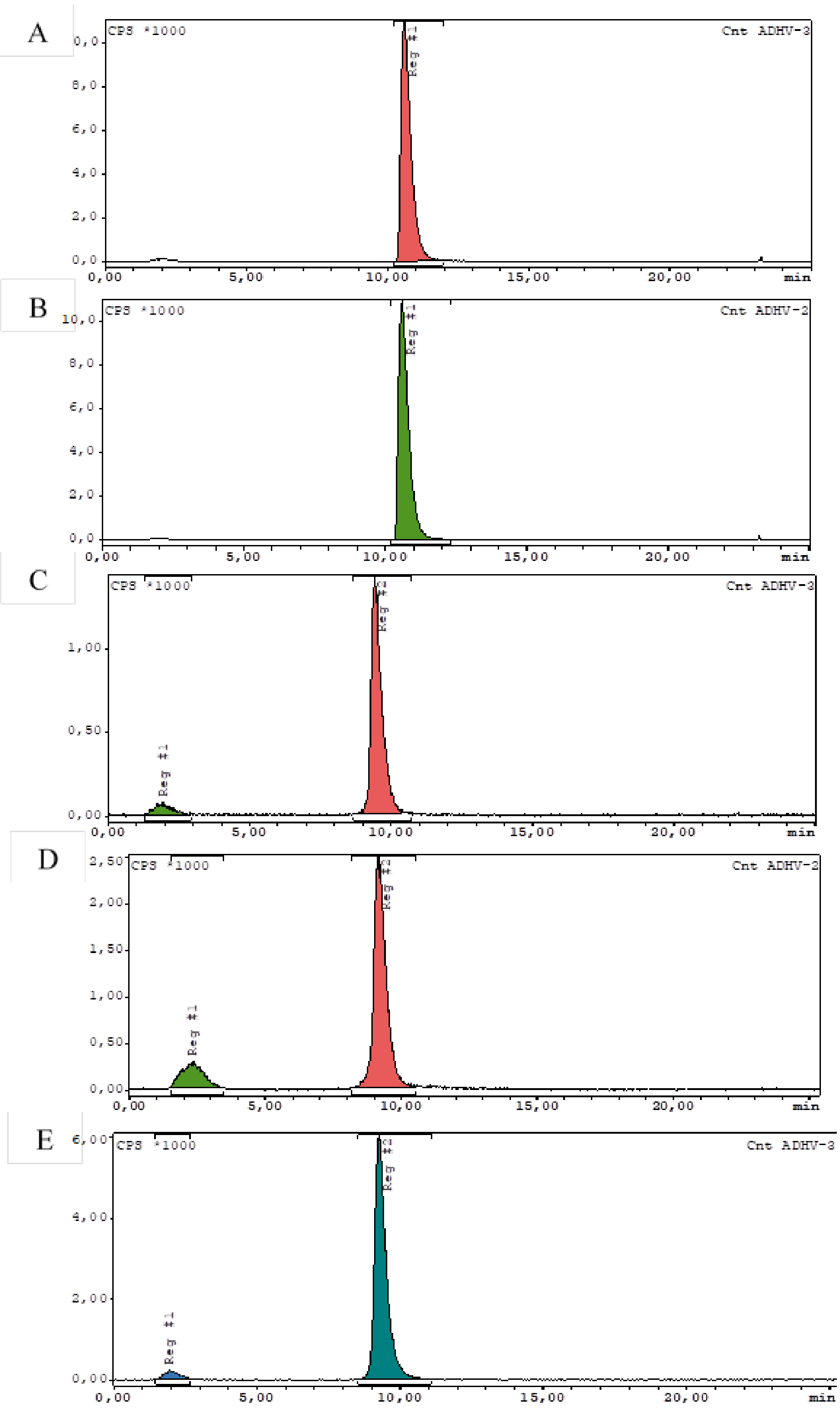

Figure S1: Radiometabolite analysis in plasma and urine ***A)*** *Radiochromatogram of rat plasma sample spiked with [^{18}F]AlF-NETA-TATE;* ***B)*** *Radiochromatogram of rat urine sample spiked with [^{18}F]AlF-NETA-TATE* ***C)*** *Radiochromatogram of plasma sample 10 min. post-*

injection; ***D)*** *Radiochromatogram of plasma sample 30 min. post-injection;* ***E)*** *Radiochromatogram of urine sample 30 min. post-injection; More than 97% of fluorine-18 corresponded to the parent tracer in 10 min and 30 min post injection in plasma and urine samples respectively. 84% of the intact radiotracer was observed 30 min post injection in plasma.*

Time (minutes)	A %	B %	Flowrate (mL/min)
0	99	1	0.5
4	99	1	0.5
4.1	99	1	1
14	10	90	1
17	10	90	1
17.1	10	90	0.5
25	99	1	0.5

Table S5: Gradient used for radiometabolite analysis *Mobile phase A (ammonium acetate 0.05M pH 5.5), mobile phase B (acetonitrile).*

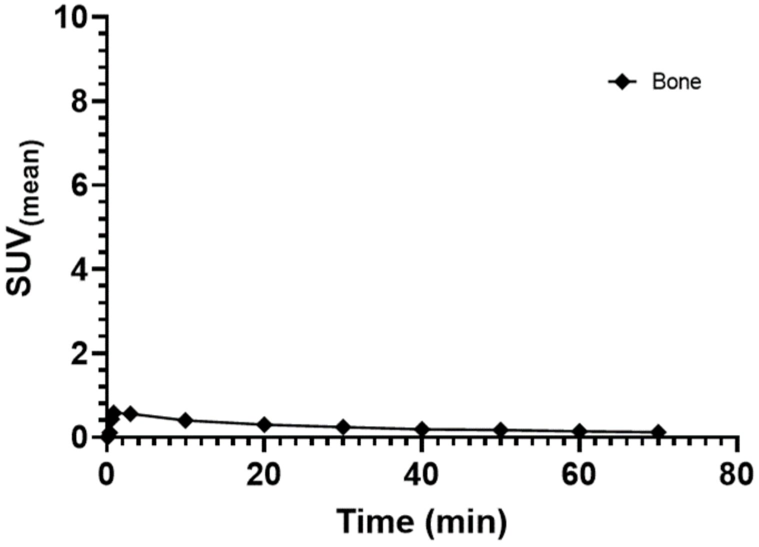

Figure S2: Bone uptake of [^{18}F]AlF-3p-*C*-NETA-TATE in rats. *Time activity curve (TAC) of bone in naïve animals (n=3).*
